# Supplementary material for: Attentional Deficits Following Preterm Birth: A Systematic Review and Meta-Analysis
Source: Brain Sci. 2025 Oct 16;15(10):1115. doi: 10.3390/brainsci15101115 (PMC12564378; doi:10.3390/brainsci15101115)
Supplement: Supplementary file 1 [file brainsci-15-01115-s001.zip › brainsci-3858910-supplementary.pdf]

Supplementary Table S1: PRISMA 2020 checklist

Section & Topic	Item #	Checklist item	Page #[1]
**TITLE**			
Title	1	Identify the report as a systematic review.	1
**ABSTRACT**			
Abstract	2	See the PRISMA 2020 for Abstracts checklist.	1
**INTRODUCTION**			
Rationale	3	Describe the rationale for the review in the context of existing knowledge.	2
Objectives	4	Provide an explicit statement of the objective(s) or question(s) the review addresses.	2
**METHODS**			
Eligibility criteria	5	Specify the inclusion and exclusion criteria for the review and how studies were grouped for the syntheses.	3
Information sources	6	Specify all databases, registers, websites, organisations, reference lists and other sources searched or consulted to identify studies. Specify the date when each source was last searched or consulted.	3
Search strategy	7	Present the full search strategies for all databases, registers and websites, including any filters and limits used.	3
Selection process	8	Specify the methods used to decide whether a study met the inclusion criteria of the review, including how many reviewers screened each record and each report retrieved, whether they worked independently, and if applicable, details of automation tools used in the process.	3
Data collection process	9	Specify the methods used to collect data from reports, including how many reviewers collected data from each report, whether they worked independently, any processes for obtaining or confirming data from study investigators, and if applicable, details of automation tools used in the process.	3
Data items	10a	List and define all outcomes for which data were sought. Specify whether all results that were compatible with each outcome domain in each study were sought (e.g. for all measures, time points, analyses), and if not, the methods used to decide which results to collect.	3<br>Table 1
	10b	List and define all other variables for which data were sought (e.g. participant and intervention characteristics, funding sources). Describe any assumptions made about any missing or unclear information.	3<br>Table 1
Study risk of bias assessment	11	Specify the methods used to assess risk of bias in the included studies, including details of the tool(s) used, how many reviewers assessed each study and whether they worked independently, and if applicable, details of automation tools used in the process.	3<br>Table S2
Effect measures	12	Specify for each outcome the effect measure(s) (e.g. risk ratio, mean difference) used in the synthesis or presentation of results.	3
Synthesis methods	13a	Describe the processes used to decide which studies were eligible for each synthesis (e.g. tabulating the study intervention characteristics and comparing against the planned groups for each synthesis (item #5)).	3
	13b	Describe any methods required to prepare the data for presentation or synthesis, such as handling of missing summary statistics, or data conversions.	3
	13c	Describe any methods used to tabulate or visually display results of individual studies and syntheses.	3
	13d	Describe any methods used to synthesize results and provide a rationale for the choice(s). If meta-analysis was performed, describe the model(s), method(s) to identify the presence and extent of statistical heterogeneity, and software	3

Section & Topic	Item #	Checklist item	Page #[1]
		package(s) used.	
	13e	Describe any methods used to explore possible causes of heterogeneity among study results (e.g. subgroup analysis, meta-regression).	N/A
	13f	Describe any sensitivity analyses conducted to assess robustness of the synthesized results.	2
Reporting bias assessment	14	Describe any methods used to assess risk of bias due to missing results in a synthesis (arising from reporting biases).	3
Certainty assessment	15	Describe any methods used to assess certainty (or confidence) in the body of evidence for an outcome.	N/A
**RESULTS**			
Study selection	16a	Describe the results of the search and selection process, from the number of records identified in the search to the number of studies included in the review, ideally using a flow diagram.	4<br>Figure 1
	16b	Cite studies that might appear to meet the inclusion criteria, but which were excluded, and explain why they were excluded.	Table S4
Study characteristics	17	Cite each included study and present its characteristics.	Table 1
Risk of bias in studies	18	Present assessments of risk of bias for each included study.	Table S2
Results of individual studies	19	For all outcomes, present, for each study: (a) summary statistics for each group (where appropriate) and (b) an effect estimate and its precision (e.g. confidence/credible interval), ideally using structured tables or plots.	6-7<br>Figure 2<br>Figure 3
Results of syntheses	20a	For each synthesis, briefly summarise the characteristics and risk of bias among contributing studies.	4
	20b	Present results of all statistical syntheses conducted. If meta-analysis was done, present for each the summary estimate and its precision (e.g. confidence/credible interval) and measures of statistical heterogeneity. If comparing groups, describe the direction of the effect.	6-7<br>Figure 2<br>Figure 3
	20c	Present results of all investigations of possible causes of heterogeneity among study results.	6-7
	20d	Present results of all sensitivity analyses conducted to assess the robustness of the synthesized results.	6-7<br>Figure 2<br>Figure 3
Reporting biases	21	Present assessments of risk of bias due to missing results (arising from reporting biases) for each synthesis assessed.	6-8<br>Figure S1<br>Figure S2
Certainty of evidence	22	Present assessments of certainty (or confidence) in the body of evidence for each outcome assessed.	6-8
**DISCUSSION**			
Discussion	23a	Provide a general interpretation of the results in the context of other evidence.	8-11
	23b	Discuss any limitations of the evidence included in the review.	11
	23c	Discuss any limitations of the review processes used.	11
	23d	Discuss implications of the results for practice, policy, and future research.	11-12
**OTHER INFORMATION**			
Registration and protocol	24a	Provide registration information for the review, including register name and registration number, or state that the review was not registered.	4

Section & Topic	Item #	Checklist item	Page #[1]
	24b	Indicate where the review protocol can be accessed, or state that a protocol was not prepared.	4
	24c	Describe and explain any amendments to information provided at registration or in the protocol.	N/A
Support	25	Describe sources of financial or non-financial support for the review, and the role of the funders or sponsors in the review.	12
Competing interests	26	Declare any competing interests of review authors.	12
Availability of data, code and other materials	27	Report which of the following are publicly available and where they can be found: template data collection forms; data extracted from included studies; data used for all analyses; analytic code; any other materials used in the review.	12

This checklist was downloaded from http://www.prisma-statement.org/, and is based on the following citation: Page MJ, et al. The PRISMA 2020 statement: an updated guideline for reporting systematic reviews. BMJ 2021;372:n71. doi: 10.1136/bmj.n71.
[1]The column "Page #" refers to the page numbers of the article PDF.

Supplementary Table S2. Search strategy

Pubmed

(preterm OR premature OR pre-mature) [TIAB] AND (attention NOT ADHD) [TIAB] AND children [TIAB]

OVID (Medline, APA/PsychArticles)

((preterm OR premature OR pre-mature) AND (attention NOT ADHD) AND children).ab.

Web of Science

AB=(preterm OR premature OR pre-mature) AND AB=(attention NOT ADHD) AND AB=(children)

EMBASE

(preterm OR premature OR pre-mature):ab AND (attention NOT ADHD):ab AND children:ab

Last day of search: 02.02.2025

Supplementary Table S3: Modified risk of bias assessment scale

**Item**	**Rating**	**Score**
Selection		*Max 3*
Representativeness of preterm cohort	Truly or at least somewhat representative of preterm-born children (e.g., population-based study, all children of a predefined region/ clinic or hospital/ or group of hospitals)	1
	Selected group of preterm-born infants (e.g. all infants admitted to NICU) or no description of the study sample	0
Representativeness of term-born cohort	Term-born cohort was drawn from the same community (e.g. same hospital/ clinic or group of clinics or same predefined region, same birth years).	1
	Term-born cohort was drawn from a different community or no description of the cohort.	0
Ascertainment of prematurity	Secure medical records, independent validation of prematurity (e.g., hospital records, gestational age assessed using antenatal ultrasound).	1
	Parental self-report or no description of the ascertainment of prematurity (only in studies that were not conducted by a hospital research group)	0
Comparability		*Max 2*
Comparability of age	Study controls for age at testing (either corrected or uncorrected)	1
	Study merely presents a statistical test describing no statistical difference of cohorts or no description of age at testing	0
Comparability of other factors	Study controls for at least one other factor: sex, socioeconomic status, maternal age, maternal education	1
	There are significant differences in all of the above-mentioned parameters.	0
Outcome		*Max 2*
Blinded assessment	Researchers were blind to the cohort (preterm or full-term).	1
	Researcher were aware of the cohort, or no description.	0
Data loss	Complete follow-up (all subjects accounted for), or subjects lost to follow-up is unlikely to produce a bias (less than 25%), or description of those lost to follow-up is provided.	1
	More than 25% of subjects are lost to follow-up, and no description of the reasons for those lost to follow-up is provided.	0

Supplementary Table S4: Quality assessment of eligible studies

	Selection			Comparability		Outcome		Total score
	Item 1	Item 2	Item 3	Item 1	Item 2	Item 1	Item 2	max 7
Anderson et al., 2011	+	+	+	+	+	+	+	7
Bayless & Stevenson, 2007	+	-	+	+	+	-	+	5
Begega et al. 2010	-	-	+	-	-	+	-	2
Cserjesi et al. 2012	+	+	+	+	+	+	+	7
Delane et al. 2017	+	-	+	-	-	-	-	2
Giordano et al. 2017	+	-	+	+	+	+	+	6
Hagmann-von Arx et al. 2014	-	-	+	+	+	-	+	4
Jaeger et al.	-	-	+	+	+	-	-	3
Ji et al. 2024	+	+	-	+	+	+	+	6
Lean et al.	-	-	+	+	+	+	+	5
Mulder et al.	+	-	+	+	+	-	+	5
Murray et al.	+	-	+	+	+	+	+	6
Potharst et al.	-	-	+	+	+	+	-	4
Rose et al.	-	+	+	+	+	-	-	4
Sejer et al.	+	+	+	+	-	-	+	5
Shum et al.	+	-	+	+	+	-	+	5
Tinelli et al.	-	-	-	+	+	-	+	3
van Baar et al.	+	-	-	+	-	-	-	2

Supplementary Figure

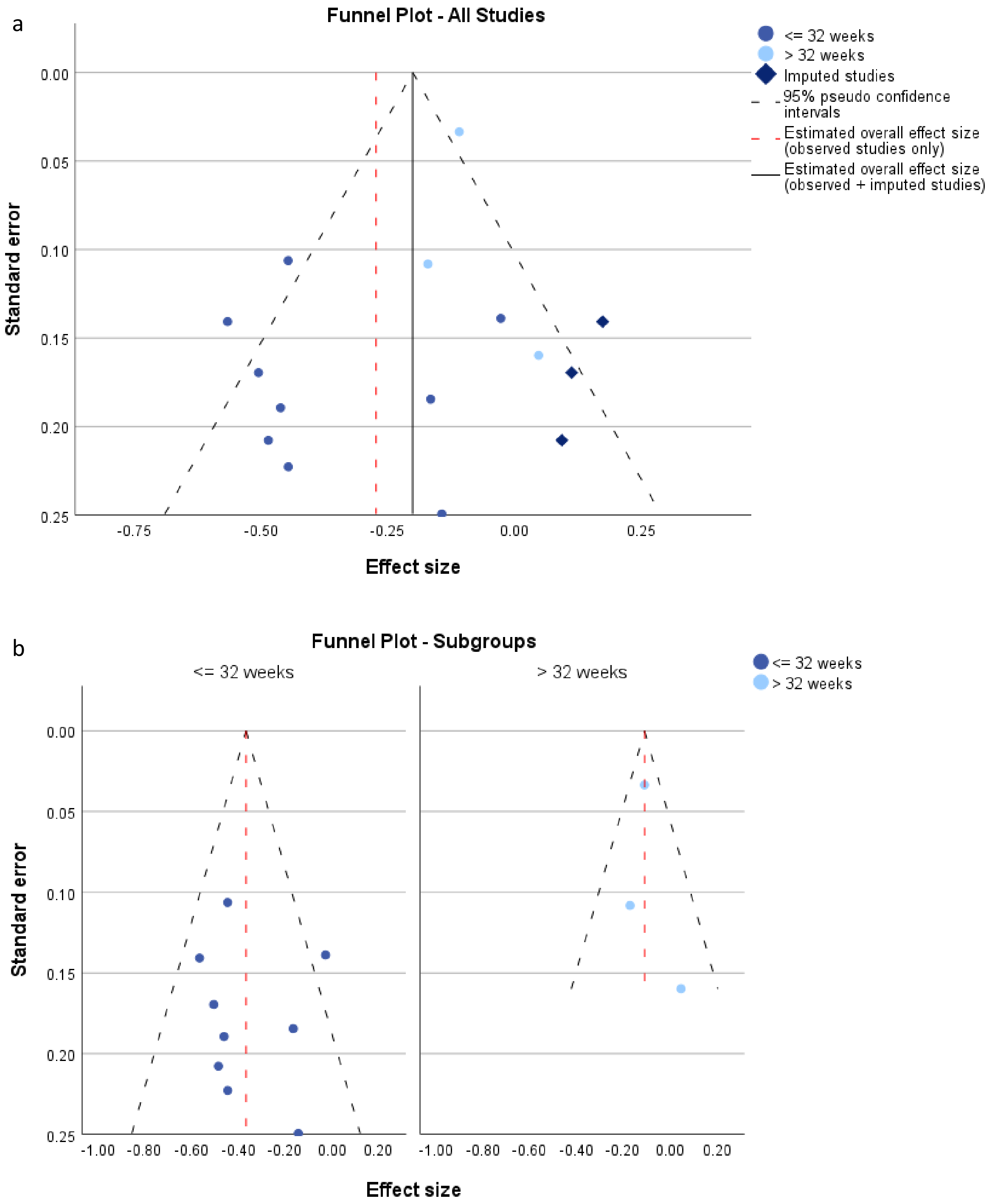

Supplementary Figure S1: Funnel plots for included studies on selective attention. a) Funnel plot for all included studies. b) Funnel plots separated by gestational age subgroups. The funnel plot for all studies looks slightly asymmetric. However, separated by subgroups, the funnel plots look more symmetric. Therefore, we assume differences in selective attention performances for the two subgroups.

a

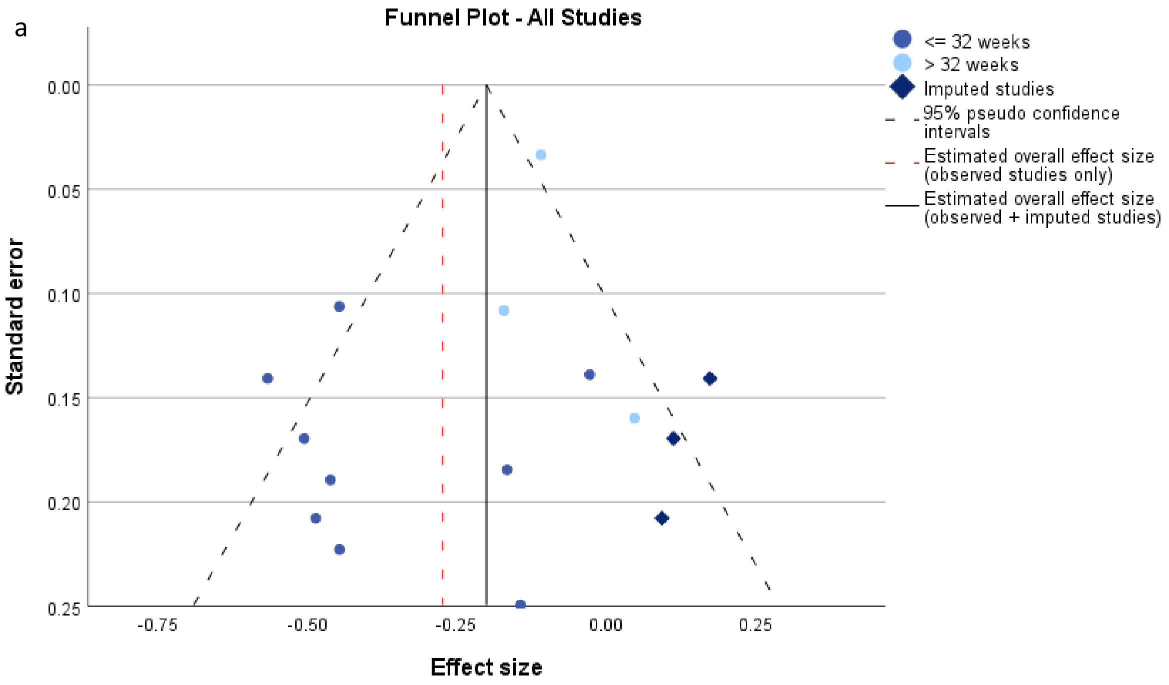

b

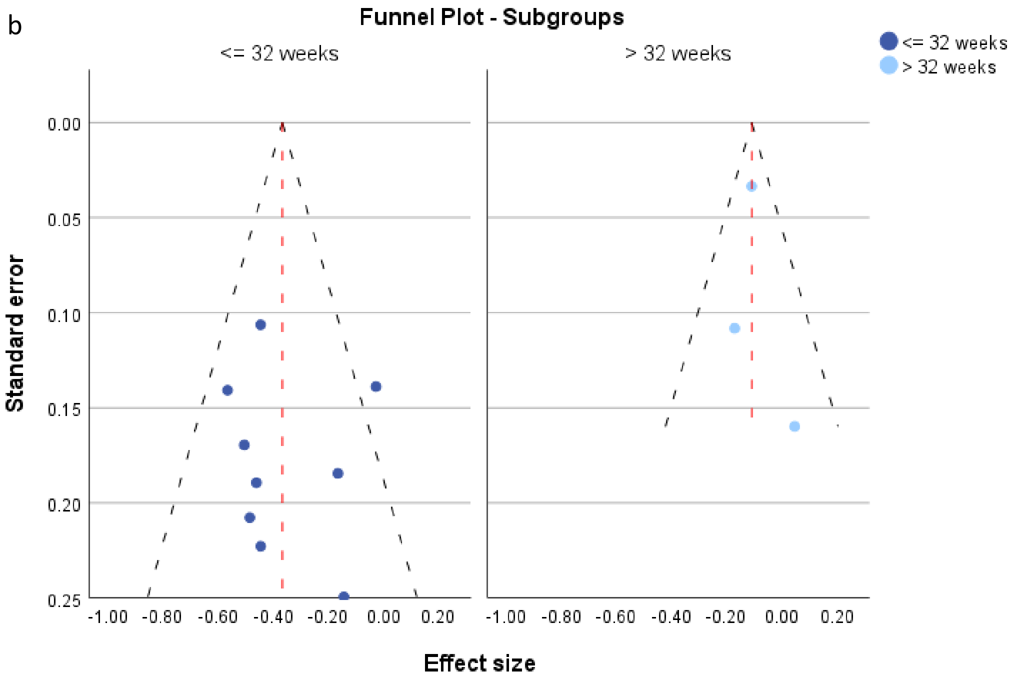

Supplementary Figure S2: Funnel plots for included studies on selective attention. a) Funnel plot for all included studies. b) Funnel plots separated by gestational age subgroups. The funnel plot for all studies looks slightly asymmetric. However, separated by subgroups, the funnel plots look more symmetric. Therefore, we assume differences in selective attention performances for the two subgroups.

a

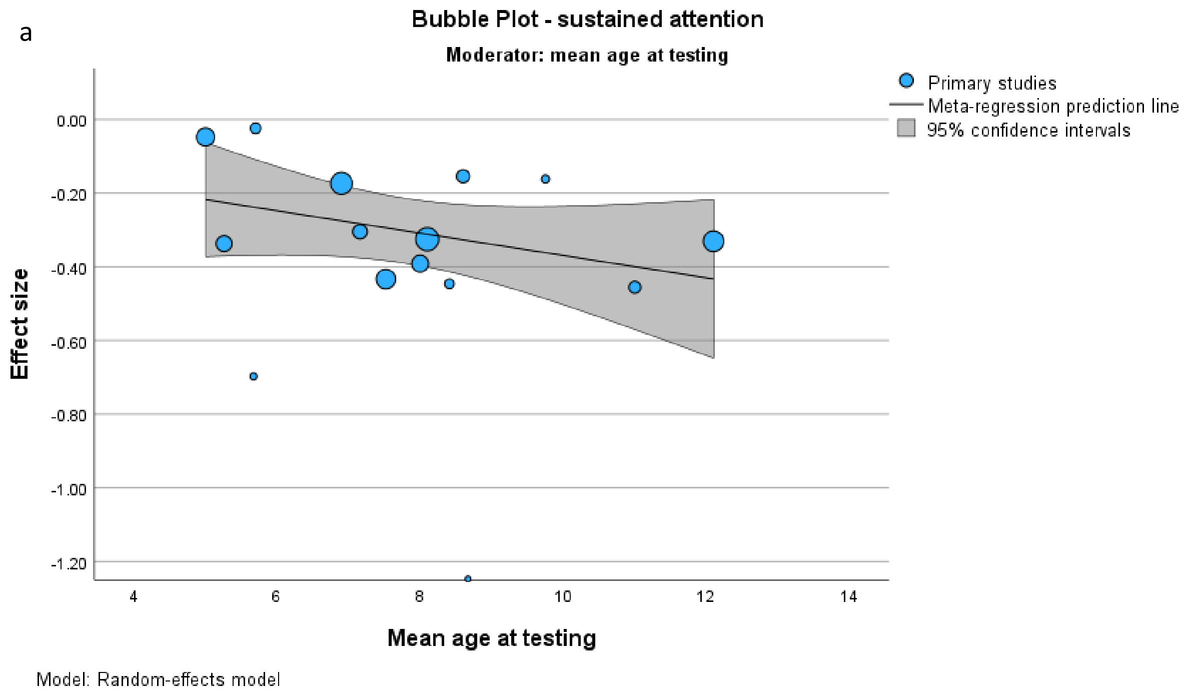

b

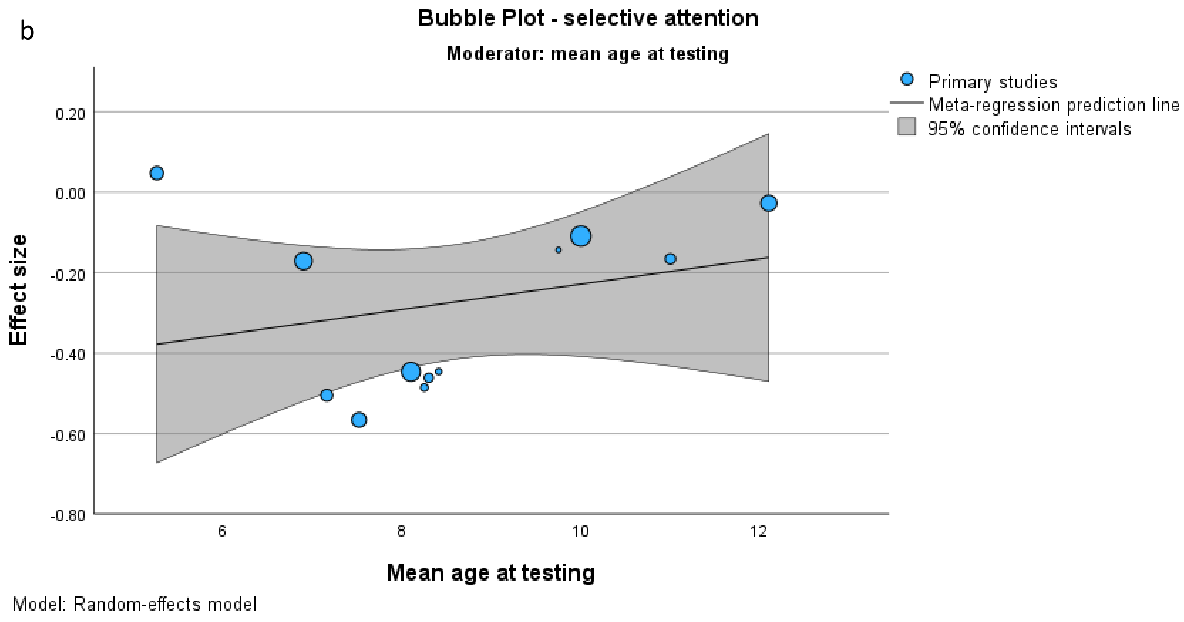

Supplementary Figure S3: Bubble plots describing the meta-regression of the impact of age at testing on a) sustained attention and b) selective attention.

Supplementary Table S5: List of studies that were excluded after full-text screening (n=273) with brief reasons.

Author	Year	Title	Journal	Exclusion reason
Aarnoudse-Moens et al.[1]	2018	Executive Function Computerized Training in Very Preterm-Born Children: A Pilot Study	GAMES FOR HEALTH JOURNAL	no control group
Aarnoudse-Moens et al.[2]	2013	Executive Function and IQ Predict Mathematical and Attention Problems in Very Preterm Children	PLOS ONE	no sustained/selective attention score
Abernethy et al. [3]	2004	Caudate and hippocampal volumes, intelligence, and motor impairment in 7-year-old children who were born preterm	PEDIATRIC RESEARCH	no sustained/selective attention score
Agarwal et al.[4]	2021	Academic school readiness in children born very preterm and associated risk factors	EARLY HUMAN DEVELOPMENT	no sustained/selective attention score
Alonso-Lopez et al.[5]	2024	Respiratory, cardio-metabolic and neurodevelopmental long-term outcomes of moderate to late preterm birth: not just a near term-population. A follow-up study	FRONTIERS IN MEDICINE	no sustained/selective attention score
Anderson et al.[6]	2018	Long-Term Academic Functioning Following Cogmed Working Memory Training for Children Born Extremely Preterm: A Randomized Controlled Trial	JOURNAL OF PEDIATRICS	no control group
Anderson et al. [7]	2003	Victorian Infant Collaborative Study Group Neurobehavioral Outcomes of School-Age Children Born Extremely Low Birth Weight or Very Preterm in the 1990s	JAMA	no sustained/selective attention score
Andersson et al.[8]	2023	Meaningful everyday life situations from the perspective of children born preterm: A photo-elicitation interview study with six-year-old children	PLOS ONE	no sustained/selective attention score
Arhan et al.[9]	2017	Regional brain volume reduction and cognitive outcomes in preterm children at low risk at 9 years of age	CHILDS NERVOUS SYSTEM	no sustained/selective attention score
Ataman-Devrim et al.[10]	2024	Neonatal risk and coordinated joint attention episodes with mothers and fathers relate to language skills of preterm children aged 2-4 years	COGNITIVE DEVELOPMENT	age group too young/too old
Ataman-Devrim et al.[11]	2024	Preterm toddlers' joint attention characteristics during dyadic interactions with their mothers and fathers compared to full-term toddlers at age 2 years	INFANT BEHAVIOR & DEVELOPMENT	age group too young/too old
Baron et al.[12]	2011	Neuropsychological and Behavioral Outcomes of Extremely Low Birth Weight at Age Three	DEVELOPMENTAL NEUROPSYCHOLOGY	focused on birth weight, not gestational age
Baron et al.[13]	2009	Age-appropriate early school age neurobehavioral outcomes of extremely preterm birth without severe intraventricular hemorrhage: a single center experience	Early Hum Dev	no control group
Baron et al.[14]	2009	Visuospatial and verbal fluency relative deficits in 'complicated' late-preterm preschool children	EARLY HUMAN DEVELOPMENT	no sustained/selective attention score

Beunders et al.[15]	2021	Early visuospatial attention and processing and related neurodevelopmental outcome at 2 years in children born very preterm	PEDIATRIC RESEARCH	age group too young/too old
Bijlsma et al.[16]	2024	Visual attention and processing function in relation to executive functioning in very preterm-born children aged 3 years: a prospective cohort study	EUROPEAN JOURNAL OF PEDIATRICS	age group too young/too old
Bogicevic et al.[17]	2021	Exploring predictors at toddler age of distinct profiles of attentional functioning in 6-year-old children born moderate-to-late preterm and full term	PLOS ONE	multiple publication of an included dataset
Bogicevic et al.[18]	2021	Individual Attention Patterns in Children Born Very Preterm and Full Term at 7 and 13 Years of Age	JOURNAL OF THE INTERNATIONAL NEUROPSYCHOLOGICAL SOCIETY	multiple publication of an included dataset
Bogicevic et al.[19]	2019	Toddler skills predict moderate-to-late preterm born children's cognition and behaviour at 6 years of age	PLOS ONE	no sustained/selective attention score
Bogicevic et al.[20]	2020	Distinct Profiles of Attention in Children Born Moderate-to-Late Preterm at 6 Years	JOURNAL OF PEDIATRIC PSYCHOLOGY	no sustained/selective attention score
Bolk et al.[21]	2018	Developmental Coordination Disorder and Its Association With Developmental Comorbidities at 6.5 Years in Apparently Healthy Children Born Extremely Preterm	JAMA PEDIATRICS	no sustained/selective attention score
Bora et al.[22]	2014	Neonatal cerebral morphometry and later risk of persistent inattention/hyperactivity in children born very preterm	JOURNAL OF CHILD PSYCHOLOGY AND PSYCHIATRY	ADHD
Botellero et al.[23]	2017	A longitudinal study of associations between psychiatric symptoms and disorders and cerebral gray matter volumes in adolescents born very preterm	BMC PEDIATRICS	ADHD
Breeman et al.[24]	2018	Infant regulatory problems, parenting quality and childhood attention problems	EARLY HUMAN DEVELOPMENT	no control group
Breeman et al.[25]	2016	Attention problems in very preterm children from childhood to adulthood: the Bavarian Longitudinal Study	JOURNAL OF CHILD PSYCHOLOGY AND PSYCHIATRY	no sustained/selective attention score
Brogan et al.[26]	2014	Inattention in very preterm children: implications for screening and detection	ARCHIVES OF DISEASE IN CHILDHOOD	ADHD
Brown et al.[27]	2023	Early parenting behaviour is associated with complex attention outcomes in middle to late childhood in children born very preterm	CHILD NEUROPSYCHOLOGY	age group too young/too old
Brown et al.[28]	2023	Motor performance and attention outcomes in children born very preterm	DEVELOPMENTAL MEDICINE AND CHILD NEUROLOGY	multiple publication of an included dataset

Bucci et al. [29]	2022	Immature brain structures were associated with poorer eye movement performance at 8 years of age in preterm born children	ACTA PAEDIATRICA	no sustained/selective attention score
Bul et al.[30]	2012	Behavior Problems in Relation to Sustained Selective Attention Skills of Moderately Preterm Children	JOURNAL OF DEVELOPMENTAL AND PHYSICAL DISABILITIES	no group scores reported
Burnett et al.[31]	2019	Exploring the Preterm Behavioral Phenotype in Children Born Extremely Preterm	JOURNAL OF DEVELOPMENTAL AND BEHAVIORAL PEDIATRICS	no sustained/selective attention score
Butcher et al.[32]	2009	The quality of preterm infants' spontaneous movements: an early indicator of intelligence and behaviour at school age	JOURNAL OF CHILD PSYCHOLOGY AND PSYCHIATRY	no sustained/selective attention score
Cainelli et al.[33]	2021	Neonatal spectral EEG is prognostic of cognitive abilities at school age in premature infants without overt brain damage	EUROPEAN JOURNAL OF PEDIATRICS	no control group
Camerota et al.[34]	2024	Trajectories of attention problems in preschoolers born very preterm	JOURNAL OF CHILD PSYCHOLOGY AND PSYCHIATRY	no sustained/selective attention score
Camerota et al.[35]	2024	Epigenome-wide association study identifies neonatal DNA methylation associated with two-year attention problems in children born very preterm	TRANSLATIONAL PSYCHIATRY	no sustained/selective attention score
Campbell et al.[36]	2015	How do you think she feels? Vulnerability in empathy and the role of attention in school-aged children born extremely preterm	BRITISH JOURNAL OF DEVELOPMENTAL PSYCHOLOGY	no sustained/selective attention score
Caravale et al.[37]	2005	Cognitive development in low risk preterm infants at 3-4 years of life	ARCHIVES OF DISEASE IN CHILDHOOD-FETAL AND NEONATAL EDITION	age group too young/too old
Caravale et al.[38]	2017	Sleep Characteristics and Temperament in Preterm Children at Two Years of Age	JOURNAL OF CLINICAL SLEEP MEDICINE	age group too young/too old
Caravale et al.[39]	2012	Change in cognitive abilities over time during preschool age in low risk preterm children	EARLY HUMAN DEVELOPMENT	no sustained/selective attention score
Cherkes-Julkowski et al.[40]	1999	Self-organization of mother-child instructional dyads and later attention disorder	JOURNAL OF LEARNING DISABILITIES	before 2000
Chin et al.[41]	2023	Correlation Analysis of Attention and Intelligence of Preterm Infants at Preschool Age: A Premature Cohort Study	INTERNATIONAL JOURNAL OF ENVIRONMENTAL RESEARCH AND PUBLIC HEALTH	age group too young/too old
Cohen et al.[42]	1983	Prediction of five-year Stanford-Binet scores in preterm infants	CHILD DEVELOPMENT	before 2000

Coratti et al.[43]	2024	Assessment of early attention in an Italian cohort of preschooler preterm children using the Early Childhood Attention Battery	EUROPEAN JOURNAL OF PEDIATRICS	age group too young/too old
Cosentino-Rocha et al.[44]	2014	Effects of preterm birth and gender on temperament and behavior in children	INFANT BEHAVIOR & DEVELOPMENT	no sustained/selective attention score
Crockett et al.[45]	2022	Education Outcomes of Children Born Late Preterm: A Retrospective Whole-Population Cohort Study	MATERNAL AND CHILD HEALTH JOURNAL	no sustained/selective attention score
Crowther et al.[46]	2007	Outcomes at 2 years of age after repeat doses of antenatal corticosteroids	NEW ENGLAND JOURNAL OF MEDICINE	no sustained/selective attention score
Cserjesi et al.[47]	2012	Patterns of functioning and predictive factors in children born moderately preterm or at term	DEVELOPMENTAL MEDICINE AND CHILD NEUROLOGY	multiple publication of an included dataset
Cusin Lamonica et al.[48]	2018	Communicative performance and vocabulary domain in preschool preterm infants	JOURNAL OF APPLIED ORAL SCIENCE	no sustained/selective attention score
Dai et al.[49]	2020	Relationships between intelligence, executive function and academic achievement in children born very preterm	EARLY HUMAN DEVELOPMENT	no control group
Danks et al.[50]	2017	Are behaviour problems in extremely low-birthweight children related to their motor ability?	ACTA PAEDIATRICA	no sustained/selective attention score
Datin-Dorriere et al.[51]	2021	The forest, the trees, and the leaves in preterm children: the impact of prematurity on a visual search task containing three-level hierarchical stimuli	EUROPEAN CHILD & ADOLESCENT PSYCHIATRY	no sustained/selective attention score
de Jong et al.[52]	2015	Attention capacities of preterm and term born toddlers: A multi-method approach	EARLY HUMAN DEVELOPMENT	age group too young/too old
de Kieviet et al.[53]	2014	A crucial role for white matter alterations in interference control problems of very preterm children	PEDIATRIC RESEARCH	multiple publication of an included dataset
de Kieviet et al.[54]	2012	Attention Problems of Very Preterm Children Compared with Age-Matched Term Controls at School-Age	JOURNAL OF PEDIATRICS	no sustained/selective attention score
De Schuymer et al.[55]	2012	Gaze aversion during social interaction in preterm infants: A function of attention skills?	INFANT BEHAVIOR & DEVELOPMENT	age group too young/too old
Dean et al.[56]	2021	Longitudinal assessment of social cognition in infants born preterm using eye-tracking and parent-child play	INFANT AND CHILD DEVELOPMENT	no sustained/selective attention score
Della Rosa et al.[57]	2021	The effects of the functional interplay between the Default Mode and Executive Control Resting State Networks on cognitive outcome in preterm born infants at 6 months of age	BRAIN AND COGNITION	no sustained/selective attention score
Delobel-Ayoub et al.[58]	2009	Behavioral Problems and Cognitive Performance at 5 Years of Age After Very Preterm Birth: The EPIPAGE Study	PEDIATRICS	no sustained/selective attention score

den Heijer et al.[59]	2024	Neurocognitive outcomes in moderately preterm born adolescents	EARLY HUMAN DEVELOPMENT	age group too young/too old
Dilworth-Bart et al.[60]	2010	Maternal Scaffolding and Preterm Toddlers' Visual-Spatial Processing and Emerging Working Memory	JOURNAL OF PEDIATRIC PSYCHOLOGY	age group too young/too old
do Espirito Santo et al.[61]	2009	Cognitive and behavioral status of low birth weight preterm children raised in a developing country at preschool age	JORNAL DE PEDIATRIA	no sustained/selective attention score
Domellof et al.[62]	2020	Risk for Behavioral Problems Independent of Cognitive Functioning in Children Born at Low Gestational Ages	FRONTIERS IN PEDIATRICS	no sustained/selective attention score
Dotinga et al.[63]	2016	Longitudinal Growth and Neuropsychological Functioning at Age 7 in Moderate and Late Preterms	PEDIATRICS	no control group
Downes et al.[64]	2018	Visual attention control differences in 12-month-old preterm infants	INFANT BEHAVIOR & DEVELOPMENT	age group too young/too old
Downey et al.[65]	2015	Antenatal and Early Postnatal Antecedents of Parent-Reported Attention Problems at 2 Years of Age	JOURNAL OF PEDIATRICS	ADHD
Doyle et al.[66]	2014	School-age Outcomes of Very Preterm Infants After Antenatal Treatment With Magnesium Sulfate vs Placebo	JAMA-JOURNAL OF THE AMERICAN MEDICAL ASSOCIATION	intervention study
Dueker et al.[67]	2017	Early Developmental Outcomes Predicted by Gestational Age From 35 to 41 Weeks	OBSTETRICAL & GYNECOLOGICAL SURVEY	no sustained/selective attention score
Dupin et al.[68]	2000	Auditory attention processing in 5-year-old children born preterm: evidence from event-related potentials	DEVELOPMENTAL MEDICINE AND CHILD NEUROLOGY	no sustained/selective attention score
Einaudi et al.[69]	2008	Neuropsychological screening of a group of preterm twins: comparison with singletons	CHILDS NERVOUS SYSTEM	ADHD
El-Mazahi et al.[70]	2014	Cognitive and behavioural outcome of preterm versus full term infants, at school entry age	Trends in Medical Research	no sustained/selective attention score
Ernstad et al.[71]	2023	Preimplantation genetic testing and child health: a national register-based study	HUMAN REPRODUCTION	no sustained/selective attention score
Eryigit-Madzwamuse et al.[72]	2015	Attention problems in relation to gestational age at birth and smallness for gestational age	EARLY HUMAN DEVELOPMENT	no control group
Espy et al.[73]	2007	Perinatal pH and neuropsychological outcomes at age 3 years in children born preterm: An exploratory study	DEVELOPMENTAL NEUROPSYCHOLOGY	age group too young/too old
Espy et al.[74]	2002	Executive functions in preschool children born preterm: Application of cognitive neuroscience paradigms	CHILD NEUROPSYCHOLOGY	no sustained/selective attention score
Fan et al.[75]	2013	Cognition, behavior and social competence of preterm low birth weight children at school age	CLINICS	no sustained/selective attention score

Farooqi et al.[76]	2013	Behaviours related to executive functions and learning skills at 11 years of age after extremely preterm birth: a Swedish national prospective follow-up study	ACTA PAEDIATRICA	age group too young/too old
Filippa et al.[77]	2024	Effect of an early music intervention on emotional and neurodevelopmental outcomes of preterm infants at 12 and 24 months	FRONTIERS IN PSYCHOLOGY	no sustained/selective attention score
Fjørtoft et al.[78]	2015	Adaptive behavior in 10-11 year old children born preterm with a very low birth weight (VLBW)	EUROPEAN JOURNAL OF PEDIATRIC NEUROLOGY	no sustained/selective attention score
Foulder-Hughes et al.[79]	2003	Motor, cognitive, and behavioural disorders in children born very preterm	DEVELOPMENTAL MEDICINE AND CHILD NEUROLOGY	no sustained/selective attention score
Frye et al.[80]	2010	Superior longitudinal fasciculus and cognitive dysfunction in adolescents born preterm and at term	DEVELOPMENTAL MEDICINE AND CHILD NEUROLOGY	age group too young/too old
Garcia-Bermudez et al.[81]	2019	Improvement of executive functions after the application of a neuropsychological intervention program (PEFEN) in pre-term children	CHILDREN AND YOUTH SERVICES REVIEW	age group too young/too old
Geldof et al.[82]	2013	Visual search and attention in five-year-old very preterm/very low birth weight children	EARLY HUMAN DEVELOPMENT	no sustained/selective attention score
Geva et al.[83]	2016	Neonatal Sleep Predicts Attention Orienting and Distractibility	JOURNAL OF ATTENTION DISORDERS	age group too young/too old
Gillenson et al.[84]	2023	A Preliminary Study of Executive Functioning in Preterm-Born Children: A Bilingual Advantage	ADVANCES IN NEONATAL CARE	no control group
Gire et al.[85]	2023	Cognitive Training for Visuospatial Processing in Children Aged 5½ to 6 Years Born Very Preterm With Working Memory Dysfunction: A Randomized Clinical Trial	JAMA NETWORK OPEN	no control group
Gire et al.[86]	2019	Quality of life of extremely preterm school-age children without major handicap: a cross-sectional observational study	ARCHIVES OF DISEASE IN CHILDHOOD	no sustained/selective attention score
Gomaa et al.[87]	2022	Association of Pediatric Buccal Epigenetic Age Acceleration With Adverse Neonatal Brain Growth and Neurodevelopmental Outcomes Among Children Born Very Preterm With a Neonatal Infection	JAMA NETWORK OPEN	no sustained/selective attention score
Gomot et al.[88]	2007	Left temporal impairment of auditory information processing in prematurely born 9-year-old children:: An electrophysiological study	INTERNATIONAL JOURNAL OF PSYCHOPHYSIOLOGY	no sustained/selective attention score
Gould et al.[89]	2019	Can the Bayley Scales of Infant Development at 18 months predict child behaviour at 7 years?	JOURNAL OF PAEDIATRICS AND CHILD HEALTH	no sustained/selective attention score
Griffiths et al.[90]	2013	fMRI: blood oxygen level-dependent activation during a working memory-selective attention task in children born extremely preterm	PEDIATRIC RESEARCH	age group too young/too old

Griffiths et al.[91]	2014	Association between brain activation (fMRI), cognition and school performance in extremely preterm and term born children	SCANDINAVIAN JOURNAL OF PSYCHOLOGY	no sustained/selective attention score
Grunewaldt et al.[92]	2016	Computerized working memory training has positive long-term effect in very low birthweight preschool children	DEVELOPMENTAL MEDICINE AND CHILD NEUROLOGY	focused on birth weight, not gestational age
Grunewaldt et al.[93]	2013	Working Memory Training Improves Cognitive Function in VLBW Preschoolers	PEDIATRICS	no control group
Grunewaldt et al.[94]	2014	Follow-up at age 10 years in ELBW children - Functional outcome, brain morphology and results from motor assessments in infancy	EARLY HUMAN DEVELOPMENT	no sustained/selective attention score
Hall et al.[95]	2012	Gender distinctive impacts of prematurity and small for gestational age (SGA) on age-6 attention problems	CHILD AND ADOLESCENT MENTAL HEALTH	no sustained/selective attention score
Hebert-Myers et al.[96]	2006	The Importance of Language, Social, and Behavioral Skills Across Early and Later Childhood as Predictors of Social Competence With Peers.	Applied Developmental Science	no sustained/selective attention score
Hellgren et al.[97]	2013	Compromised approximate number system acuity in extremely preterm school-aged children	DEVELOPMENTAL MEDICINE AND CHILD NEUROLOGY	no sustained/selective attention score
Hemgren et al.[98]	2007	Associations of motor co-ordination and attention with motor-perceptual development in 3-year-old preterm and full-term children who needed neonatal intensive care	CHILD CARE HEALTH AND DEVELOPMENT	no sustained/selective attention score
Hille et al.[99]	1994	School performance at nine years of age in very premature and very low birth weight infants: perinatal risk factors and predictors at five years of age. Collaborative Project on Preterm and Small for Gestational Age (POPS) Infants in The Netherlands	JOURNAL OF PEDIATRICS	before 2000
Hodel et al.[100]	2017	Early executive function differences in infants born moderate-to-late preterm	EARLY HUMAN DEVELOPMENT	age group too young/too old
Hofheimer et al.[101]	2023	Assessment of Psychosocial and Neonatal Risk Factors for Trajectories of Behavioral Dysregulation Among Young Children From 18 to 72 Months of Age	JAMA NETWORK OPEN	no sustained/selective attention score
Imgrund et al.[102]	2019	Expressive Language in Preschoolers Born Preterm: Results of Language Sample Analysis and Standardized Assessment	JOURNAL OF SPEECH LANGUAGE AND HEARING RESEARCH	no sustained/selective attention score
Indredavik et al.[103]	2010	Perinatal Risk and Psychiatric Outcome in Adolescents Born Preterm With Very Low Birth Weight or Term Small for Gestational Age	JOURNAL OF DEVELOPMENTAL AND BEHAVIORAL PEDIATRICS	focused on birth weight, not gestational age
Ionio et al.[104]	2022	Cognitive, Behavioral and Socioemotional Development in a Cohort of Preterm Infants at School Age: A Cross-Sectional Study	PEDIATRIC REPORTS	no control group

Jaeger et al.[105]	2019	Reduction of the event-related potential P3 in preterm born 5-year-old healthy children	CLINICAL NEUROPHYSIOLOGY	no sustained/selective attention score
Jaekel et al.[106]	2021	Preterm children's long-term academic performance after adaptive computerized training: an efficacy and process analysis of a randomized controlled trial	PEDIATRIC RESEARCH	intervention study
Jaekel et al.[107]	2013	Poor attention rather than hyperactivity/impulsivity predicts academic achievement in very preterm and full-term adolescents	PSYCHOLOGICAL MEDICINE	no sustained/selective attention score
Jaekel et al.[108]	2016	Preterm Toddlers' Inhibitory Control Abilities Predict Attention Regulation and Academic Achievement at Age 8 Years	JOURNAL OF PEDIATRICS	no sustained/selective attention score
Janson et al.[109]	2024	Dolphin CONTINUE: a multi-center randomized controlled trial to assess the effect of a nutritional intervention on brain development and long-term outcome in infants born before 30 weeks of gestation	BMC PEDIATRICS	no sustained/selective attention score
Jin et al.[110]	2020	Long-term cognitive, executive, and behavioral outcomes of moderate and late preterm at school age	CLINICAL AND EXPERIMENTAL PEDIATRICS	no control group
Johnson et al.[111]	2011	Educational Outcomes in Extremely Preterm Children: Neuropsychological Correlates and Predictors of Attainment	DEVELOPMENTAL NEUROPSYCHOLOGY	no sustained/selective attention score
Joo et al.[112]	2015	Neuropsychological Outcomes of Preterm Birth in Children With No Major Neurodevelopmental Impairments in Early Life	ANNALS OF REHABILITATION MEDICINE-ARM	ADHD
Joseph et al.[113]	2016	Neurocognitive and Academic Outcomes at Age 10 Years of Extremely Preterm Newborns	PEDIATRICS	no control group
Kallankari et al.[114]	2015	Very preterm birth and foetal growth restriction are associated with specific cognitive deficits in children attending mainstream school	ACTA PAEDIATRICA	no sustained/selective attention score
Kaul et al.[115]	2022	Visual tracking at 4 months in preterm infants predicts 6.5-year cognition and attention	PEDIATRIC RESEARCH	ADHD
Kim et al.[116]	2023	Association of Brain Microstructure and Functional Connectivity With Cognitive Outcomes and Postnatal Growth Among Early School-Aged Children Born With Extremely Low Birth Weight	JAMA NETWORK OPEN	multiple publication of an included dataset
Kim et al.[117]	2021	Cognitive and Behavioral Outcomes of School-aged Children Born Extremely Preterm: a Korean Single-center Study with Long-term Follow-up	JOURNAL OF KOREAN MEDICAL SCIENCE	no sustained/selective attention score
Knops et al.[118]	2005	Catch-up growth up to ten years of age in children born very preterm or with very low birth weight	BMC PEDIATRICS	no sustained/selective attention score
Kochukhova et al.[119]	2022	Antenatal steroids and neurodevelopment in 12-year-old children born extremely preterm	ACTA PAEDIATRICA	no sustained/selective attention score
Kooiker et al.[120]	2019	Perinatal risk factors for visuospatial attention and processing dysfunctions at 1 year of age in children born between 26 and 32 weeks	EARLY HUMAN DEVELOPMENT	age group too young/too old

Krägeloh-Mann et al.[121]	1999	Brain lesions in preterms: origin, consequences and compensation	ACTA PAEDIATRICA	before 2000
Kuban et al.[122]	2015	The Breadth and Type of Systemic Inflammation and the Risk of Adverse Neurological Outcomes in Extremely Low Gestation Newborns	PEDIATRIC NEUROLOGY	no sustained/selective attention score
Kurpershoek et al.[123]	2016	Minor neurological dysfunction in five year old very preterm children is associated with lower processing speed	EARLY HUMAN DEVELOPMENT	no control group
Lambe et al.[124]	2006	Maternal smoking during pregnancy and school performance at age 15	EPIDEMIOLOGY	no sustained/selective attention score
Landsem et al.[125]	2015	Does An Early Intervention Influence Behavioral Development Until Age 9 in Children Born Prematurely?	CHILD DEVELOPMENT	no sustained/selective attention score
Langkamp et al.[126]	1999	Risk for later school problems in preterm children who do not cooperate for preschool developmental testing	JOURNAL OF PEDIATRICS	before 2000
Larsen et al.[127]	2023	Comparing behavioural outcomes in children born extremely preterm between 2006 and 1995: the EPICure studies	EUROPEAN CHILD & ADOLESCENT PSYCHIATRY	no sustained/selective attention score
Lawrence et al.[128]	2009	The Neural Basis of Response Inhibition and Attention Allocation as Mediated by Gestational Age	HUMAN BRAIN MAPPING	age group too young/too old
Lawson et al.[129]	1992	MATERNAL-BEHAVIOR AND INFANT ATTENTION	INFANT BEHAVIOR & DEVELOPMENT	before 2000
Lawson et al.[130]	2004	Early focused attention predicts outcome for children born prematurely	JOURNAL OF DEVELOPMENTAL AND BEHAVIORAL PEDIATRICS	no sustained/selective attention score
Lean et al.	2017	Attention and Regional Gray Matter Development in Very Preterm Children at Age 12 Years	JOURNAL OF THE INTERNATIONAL NEUROPSYCHOLOGICAL SOCIETY	age group too young/too old
Lean et al.[131]	2020	Maternal and family factors differentiate profiles of psychiatric impairments in very preterm children at age 5-years	JOURNAL OF CHILD PSYCHOLOGY AND PSYCHIATRY	no sustained/selective attention score
Lee et al.[132]	2017	Effects of working memory training on children born preterm	APPLIED NEUROPSYCHOLOGY-CHILD	age group too young/too old
Lee et al.[133]	2022	Development of cool and hot executive function deficit in children born very low birth weight with normal early development: A longitudinal cohort from aged 6 to 10	EARLY HUMAN DEVELOPMENT	no sustained/selective attention score
Lejeune et al.[134]	2019	Effects of an Early Postnatal Music Intervention on Cognitive and Emotional Development in Preterm Children at 12 and 24 Months: Preliminary Findings	FRONTIERS IN PSYCHOLOGY	age group too young/too old

Lejeune et al.[135]	2015	Emotion, attention, and effortful control in 24-month-old very preterm and full-term children	ANNEE PSYCHOLOGIQUE	age group too young/too old
Leonhardt et al.[136]	2012	Visual performance in preterm infants with brain injuries compared with low-risk preterm infants	EARLY HUMAN DEVELOPMENT	no sustained/selective attention score
Lindgren et al.[137]	2000	Event-related potential findings in healthy extremely pre-term (〈 week 29) children at age 10	CHILD NEUROPSYCHOLOGY	no sustained/selective attention score
Litt et al.[138]	2023	Impaired pulmonary function mediates inattention in young adults born extremely preterm	ACTA PAEDIATRICA	ADHD
Litt et al.[139]	2012	Academic achievement of adolescents born with extremely low birth weight	ACTA PAEDIATRICA	focused on birth weight, not gestational age
Loe et al.[140]	2013	Attention and Internalizing Behaviors in Relation to White Matter in Children Born Preterm	JOURNAL OF DEVELOPMENTAL AND BEHAVIORAL PEDIATRICS	age group too young/too old
Loe et al.[141]	2011	Behavior problems of 9-16 year old preterm children: Biological, sociodemographic, and intellectual contributions	EARLY HUMAN DEVELOPMENT	no sustained/selective attention score
Logan et al.[142]	2017	Early postnatal illness severity scores predict neurodevelopmental impairments at 10 years of age in children born extremely preterm	JOURNAL OF PERINATOLOGY	no control group
Loi et al.[143]	2017	Using Eye Movements to Assess Language Comprehension in Toddlers Born Preterm and Full Term	JOURNAL OF PEDIATRICS	no sustained/selective attention score
Lonnberg et al.[144]	2021	Secondary somatosensory cortex evoked responses and 6-year neurodevelopmental outcome in extremely preterm children	CLINICAL NEUROPHYSIOLOGY	no sustained/selective attention score
Ludyga et al.[145]	2023	Martial Arts and Cognitive Control in Children with Attention-Deficit Hyperactivity Disorder and Children Born Very Preterm: A Combined Analysis of Two Randomized Controlled Trials	MEDICINE & SCIENCE IN SPORTS & EXERCISE	ADHD
Mahurin-Smith et al.[146]	2021	Children Born Prematurely May Demonstrate Catch-Up Growth in Pre-Adolescence	LANGUAGE SPEECH AND HEARING SERVICES IN SCHOOLS	no sustained/selective attention score
Marlow et al.[147]	2007	Motor and executive function at 6 years of age after extremely preterm birth	PEDIATRICS	no sustained/selective attention score
McGrath et al.[148]	2002	Birth weight, neonatal morbidities, and school age outcomes in full-term and preterm infants.	ISSUES IN COMPREHENSIVE PEDIATRIC NURSING	no sustained/selective attention score
Mikkola et al.[149]	2005	Neurodevelopmental outcome at 5 years of age of a national cohort of extremely low birth weight infants who were born in 1996-1997	PEDIATRICS	focused on birth weight, not gestational age
Molloy et al.[150]	2017	The contribution of visual processing to academic achievement in adolescents born extremely preterm or extremely low birth weight	CHILD NEUROPSYCHOLOGY	no sustained/selective attention score

Morales et al.[151]	2013	Early precursors of low attention and hyperactivity in moderately and very preterm children at preschool age	PEDIATRIC REPORTS	no sustained/selective attention score
Morsing et al.[152]	2022	Neurodevelopmental disorders and somatic diagnoses in a national cohort of children born before 24 weeks of gestation	ACTA PAEDIATRICA	ADHD
Morsing et al.[153]	2011	Cognitive Function After Intrauterine Growth Restriction and Very Preterm Birth	PEDIATRICS	no sustained/selective attention score
Murner-Lavanchy et al.[154]	2019	Thirteen-Year Outcomes in Very Preterm Children Associated with Diffuse Excessive High Signal Intensity on Neonatal Magnetic Resonance Imaging	JOURNAL OF PEDIATRICS	age group too young/too old
Murray et al.[155]	2016	White matter abnormalities and impaired attention abilities in children born very preterm	NEUROIMAGE	multiple publication of an included dataset
Nakagawa et al.[156]	2023	Attentional differences in audiovisual face perception between full- and preterm very low birthweight toddlers	ACTA PAEDIATRICA	focused on birth weight, not gestational age
Nelson et al.[157]	2021	Comparing Face-to-Face and Online Data Collection Methods in Preterm and Full-Term Children: An Exploratory Study	FRONTIERS IN PSYCHOLOGY	no reported comparison between preterms and TB
Ni et al.[158]	2011	Executive function deficit in preschool children born very low birth weight with normal early development	EARLY HUMAN DEVELOPMENT	no sustained/selective attention score
Nobre et al.[159]	2020	Effortful control and attention as predictors of cognition in children born preterm	CLINICAL CHILD PSYCHOLOGY AND PSYCHIATRY	no control group
Ochiai et al.[160]	2015	Longitudinal study of very low birth weight infants until 9years of age; attention deficit hyperactivity and autistic features are correlated with their cognitive functions	EARLY HUMAN DEVELOPMENT	focused on birth weight, not gestational age
Odd et al.[161]	2012	Long-term cognitive outcomes of infants born moderately and late preterm	DEVELOPMENTAL MEDICINE AND CHILD NEUROLOGY	attention scores for only a part of the study sample
O'Meagher et al.[162]	2020	Parent and teacher reporting of executive function and behavioral difficulties in preterm and term children at kindergarten	APPLIED NEUROPSYCHOLOGY-CHILD	age group too young/too old
Oosterom et al.[163]	2020	Parenting Behavior at 18 Months Predicts Internalizing and Externalizing Problems at 6 Years in Moderately Preterm and Full Term Children	INTERNATIONAL JOURNAL OF ENVIRONMENTAL RESEARCH AND PUBLIC HEALTH	age group too young/too old
O'Shea et al.[164]	2014	Elevated blood levels of inflammation-related proteins are associated with an attention problem at age 24 mo in extremely preterm infants	PEDIATRIC RESEARCH	age group too young/too old
Ostgard et al.[165]	2016	Executive function relates to surface area of frontal and temporal cortex in very-low-birth-weight late teenagers	EARLY HUMAN DEVELOPMENT	age group too young/too old

Padilla et al.[166]	2023	Disrupted resting-sate brain network dynamics in children born extremely preterm	CEREBRAL CORTEX	no sustained/selective attention score
Palumbi et al.[167]	2018	Neurodevelopmental and emotional-behavioral outcomes in late-preterm infants: an observational descriptive case study	BMC PEDIATRICS	ADHD
Pascoe et al.[168]	2013	Preventing academic difficulties in preterm children: a randomised controlled trial of an adaptive working memory training intervention - IMPRINT study	BMC PEDIATRICS	intervention study
Perez-Fernandez et al.[169]	2017	Go/NoGo training improves executive functions in an 8-year-old child born preterm	REVISTA DE PSICOLOGIA CLINICA CON NINOS Y ADOLESCENTES	case study
Perricone et al.[170]	2013	Neurodevelopmental outcomes of moderately preterm birth: precursors of attention deficit hyperactivity disorder at preschool age	SPRINGER PLUS	ADHD
Perricone et al.[171]	2011	The temperament of preterm infant in preschool age	ITALIAN JOURNAL OF PEDIATRICS	no sustained/selective attention score
Petrie Thomas et al.[172]	2012	Focused attention, heart rate deceleration, and cognitive development in preterm and full-term infants	DEVELOPMENTAL PSYCHOBIOLOGY	no sustained/selective attention score
Pierrat et al.[173]	2021	Neurodevelopmental outcomes at age 5 among children born preterm: EPIPAGE-2 cohort study	BMJ-BRITISH MEDICAL JOURNAL	no sustained/selective attention score
Pizzo, R et al.[174]	2010	Attentional networks efficiency in preterm children	JOURNAL OF THE INTERNATIONAL NEUROPSYCHOLOGICAL SOCIETY	no sustained/selective attention score
Poehlmann et al.[175]	2010	The Development of Effortful Control in Children Born Preterm	JOURNAL OF CLINICAL CHILD AND ADOLESCENT PSYCHOLOGY	no sustained/selective attention score
Purdy et al.[176]	2013	A Psychoneuroimmunologic Examination of Cumulative Perinatal Steroid Exposures and Preterm Infant Behavioral Follow-Up	BIOLOGICAL RESEARCH FOR NURSING	no sustained/selective attention score
Rand et al.[177]	2016	Neonatal Infection and Later Neurodevelopmental Risk in the Very Preterm Infant	JOURNAL OF PEDIATRICS	ADHD
Reid et al.[178]	2019	A Population-Based Study of School Readiness Determinants in a Large Urban Public School District	MATERNAL AND CHILD HEALTH JOURNAL	no sustained/selective attention score
Reijneveld et al.[179]	2021	Stability of Executive Functioning of Moderately-Late Preterm and Full-Term Born Children at Ages 11 and 19: The TRAILS Cohort Study	INTERNATIONAL JOURNAL OF ENVIRONMENTAL RESEARCH AND PUBLIC HEALTH	age group too young/too old

Reijneveld et al.[180]	2006	Behavioural and emotional problems in very preterm and very low birthweight infants at age 5 years	ARCHIVES OF DISEASE IN CHILDHOOD-FETAL AND NEONATAL EDITION	no sustained/selective attention score
Retzler et al.[181]	2020	Using Drift Diffusion Modeling to Understand Inattentive Behavior in Preterm and Term-Born Children	NEUROPSYCHOLOGY	multiple publication of an included dataset
Retzler et al.[182]	2019	Cognitive predictors of parent-rated inattention in very preterm children: The role of working memory and processing speed	CHILD NEUROPSYCHOLOGY	no sustained/selective attention score
Reuner et al.[183]	2015	Cognitive development in 7- to 24-month-old extremely/very-to-moderately/late preterm and full-term born infants: The mediating role of focused attention.	CHILD NEUROPSYCHOLOGY	no sustained/selective attention score
Reveillon et al.[184]	2016	Response inhibition difficulties in preterm children aged 9-12 years: Relations with emotion and behavior	CHILD NEUROPSYCHOLOGY	ADHD
Réveillon et al.[185]	2013	Functional neuroimaging study of performances on a Go/No-go task in 6- to 7-year-old preterm children: Impact of intrauterine growth restriction	Neuroimage Clin	no control group
Ribeiro et al.[186]	2011	Attention problems and language development in preterm low-birth-weight children: Cross-lagged relations from 18 to 36 months	BMC PEDIATRICS	no sustained/selective attention score
Ritter et al.[187]	2014	Cognitive and behavioral aspects of executive functions in children born very preterm	CHILD NEUROPSYCHOLOGY	no sustained/selective attention score
Rose et al.[188]	2009	Information Processing in Toddlers: Continuity from Infancy and Persistence of Preterm Deficits	INTELLIGENCE	age group too young/too old
Ross et al.[189]	2022	Relationship of withdrawn, anxious/depressed and attention problems to cognitive performance in preterm children at 18 months and 36 months of age	INFANT BEHAVIOR & DEVELOPMENT	no sustained/selective attention score
Ross-Sheehy et al.[190]	2017	Visual orienting and attention deficits in 5-and 10-month-old preterm infants	INFANT BEHAVIOR & DEVELOPMENT	age group too young/too old
Roze et al.[191]	2021	Multi-domain cognitive impairments at school age in very preterm-born children compared to term-born peers	BMC PEDIATRICS	no group scores reported
Ruff HA et al.[192]	1990	Long-term stability of individual differences in sustained attention in the early years.DP - Feb 1990	CHILD DEVELOPMENT	before 2000
Rushe et al.[193]	2001	Neuropsychological outcome at adolescence of very preterm birth and its relation to brain structure	DEVELOPMENTAL MEDICINE AND CHILD NEUROLOGY	age group too young/too old
Ruys et al.[194]	2019	Neurodevelopment of children born very preterm and/or with a very low birth weight: 8-Year follow-up of a nutritional RCT	CLINICAL NUTRITION ESPEN	no control group
Ryu et al.[195]	2017	Object permanence and the development of attention capacity in preterm and term infants: an eye-tracking study	ITALIAN JOURNAL OF PEDIATRICS	no sustained/selective attention score

Sajaniemi et al.[196]	1998	Temperament profiles and their role in neurodevelopmental assessed preterm children at two years of age	EURPEAN CHILD AND ADOLESCENT PSYCHIATRY	before 2000
Salavati et al.[197]	2021	Very Preterm Early Motor Repertoire and Neurodevelopmental Outcomes at 8 Years	PEDIATRICS	no sustained/selective attention score
Samara et al.[198]	2008	Pervasive behavior problems at 6 years of age in a total-population sample of children born at ≤25 weeks of gestation	PEDIATRICS	no sustained/selective attention score
Samuelsson et al.[199]	2017	Behavioral Patterns in Adolescents Born at 23 to 25 Weeks of Gestation	PEDIATRICS	no sustained/selective attention score
Sansavini et al.[200]	2015	Dyadic co-regulation, affective intensity and infant's development at 12 months: A comparison among extremely preterm and full-term dyads	INFANT BEHAVIOR & DEVELOPMENT	no sustained/selective attention score
Schieve et al.[201]	2016	Population impact of preterm birth and low birth weight on developmental disabilities in US children	ANNALS OF EPIDEMIOLOGY	ADHD
Schmidt et al.[202]	2002	Caregiver attention-focusing and children's attention-sharing behaviours as predictors of later verbal IQ in very low birthweight children	JOURNAL OF CHILD LANGUAGE	no sustained/selective attention score
Schothorst et al.[203]	1996	Long-term behavioral sequelae of prematurity	JOURNAL OF THE AMERICAN ACADEMY OF CHILD AND ADOLESCENT PSYCHIATRY	before 2000
Schwichtenberg et al.[204]	2016	Circadian Sleep Patterns in Toddlers Born Preterm: Longitudinal Associations with Developmental and Health Concerns	JOURNAL OF DEVELOPMENTAL AND BEHAVIORAL PEDIATRICS	no sustained/selective attention score
Scott et al.[205]	2018	Trajectories of problem behaviors from 4 to 23 years in former preterm infants	INTERNATIONAL JOURNAL OF BEHAVIORAL DEVELOPMENT	age group too young/too old
Scratch et al.[206]	2015	High Postnatal Growth Hormone Levels Are Related to Cognitive Deficits in a Group of Children Born Very Preterm	JOURNAL OF CLINICAL ENDOCRINOLOGY & METABOLISM	no control group
Sekigawa-Hosozawa et al.[207]	2017	A group of very preterm children characterized by atypical gaze patterns	BRAIN & DEVELOPMENT	no sustained/selective attention score
Serenius et al.[208]	2023	Neurobehavioral symptoms in children born extremely preterm: A Swedish National Study	ACTA PAEDIATRICA	ADHD
Shaw et al.[209]	2019	Reduced Neurosteroid Exposure Following Preterm Birth and Its' Contribution to Neurological Impairment: A Novel Avenue for Preventative Therapies	FRONTIERS IN PHYSIOLOGY	no sustained/selective attention score
Shi et al.[210]	2013	A Multivariate Surface-Based Analysis of the Putamen in Premature Newborns: Regional Differences within the Ventral Striatum	PLOS ONE	no sustained/selective attention score

Shih et al.[211]	2018	Chinese handwriting performance in preterm children in grade 2	PLOS ONE	no sustained/selective attention score
Shinya et al.[212]	2022	Cognitive flexibility in 12-month-old preterm and term infants is associated with neurobehavioural development in 18-month-olds	SCIENTIFIC REPORTS	no sustained/selective attention score
Sigman et al.[213]	1986	Infant attention in relation to intellectual abilities in childhood. [References].DP - Nov 1986	Developmental Psychology	before 2000
Simon et al.[214]	2017	Post-term growth and cognitive development at 5 years of age in preterm children: Evidence from a prospective population-based cohort	PLOS ONE	no sustained/selective attention score
Smith et al.[215]	1996	The relation of medical risk and maternal stimulation with preterm infants' development of cognitive, language and daily living skills	JOURNAL OF CHILD PSYCHOLOGY AND PSYCHIATRY	before 2000
Smyrni et al.[216]	2021	Moderately and Late Preterm Infants: Short- and Long-Term Outcomes From a Registry-Based Cohort	FRONTIERS IN NEUROLOGY	no sustained/selective attention score
Snijders et al.[217]	2020	Toddlers' Language Development: The Gradual Effect of Gestational Age, Attention Capacities, and Maternal Sensitivity	INTERNATIONAL JOURNAL OF ENVIRONMENTAL RESEARCH AND PUBLIC HEALTH	no sustained/selective attention score
Squarza et al.[218]	2020	Neurodevelopmental Outcome and Adaptive Behavior in Preterm Multiples and Singletons at 1 and 2 Years of Corrected Age	FRONTIERS IN PSYCHOLOGY	ADHD
Stedall et al.[219]	2022	Thirteen-Year Outcomes of a Randomized Clinical Trial of Early Preventive Care for Very Preterm Infants and Their Parents	JOURNAL OF PEDIATRICS	age group too young/too old
Stedall et al.[220]	2023	Episodic and prospective memory difficulties in 13-year-old children born very preterm	JOURNAL OF THE INTERNATIONAL NEUROPSYCHOLOGICAL SOCIETY	no sustained/selective attention score
Stjernqvist et al.[221]	1999	Ten-year follow-up of children born before 29 gestational weeks: health, cognitive development, behaviour and school achievement	ACTA PAEDIATRICA	before 2000
Strang-Karlsson et al.[222]	2010	Slower Reaction Times and Impaired Learning in Young Adults With Birth Weight <1500 g	PEDIATRICS	age group too young/too old
Stroustrup et al.[223]	2018	Neonatal intensive care unit phthalate exposure and preterm infant neurobehavioral performance	PLOS ONE	phtalate exposure
Suikkanen et al.[224]	2021	Reaction times, learning, and executive functioning in adults born preterm	PEDIATRIC RESEARCH	age group too young/too old
Sun et al.[225]	2012	A comparison of sustained attention in very preterm and term infants	INTERNATIONAL JOURNAL OF CHILD AND ADOLESCENT HEALTH	age group too young/too old

Sun et al.[226]	2009	A comparison of executive function in very preterm and term infants at 8 months corrected age	EARLY HUMAN DEVELOPMENT	no sustained/selective attention score
Takeuchi et al.[227]	2018	Intelligence test at preschool-age predicts reading difficulty among school-aged very low birth weight infants in Japan	BRAIN & DEVELOPMENT	focused on birth weight, not gestational age
Takeuchi et al.[228]	2019	Catch-up growth and behavioral development among preterm, small-for-gestational-age children: A nationwide Japanese population-based study	BRAIN & DEVELOPMENT	no sustained/selective attention score
Talge et al.[229]	2012	Late-Preterm Birth by Delivery Circumstance and Its Association With Parent-Reported Attention Problems in Childhood	JOURNAL OF DEVELOPMENTAL AND BEHAVIORAL PEDIATRICS	age group too young/too old
Talge et al.[230]	2011	Late-Preterm Birth and Its Association With Cognitive and Socioemotional Outcomes at 6 Years of Age	OBSTETRICAL & GYNECOLOGICAL SURVEY	no sustained/selective attention score
Tanaka et al.[231]	2013	The pilot study: Sphingomyelin-fortified milk has a positive association with the neurobehavioural development of very low birth weight infants during infancy, randomized control trial	BRAIN & DEVELOPMENT	intervention study
Tanis et al.[232]	2012	Functional outcome of very preterm-born and small-for-gestational-age children at school age	PEDIATRIC RESEARCH	no control group
Thiriez et al.[233]	2015	Altered autonomic control in preterm newborns with impaired neurological outcomes	CLINICAL AUTONOMIC RESEARCH	no sustained/selective attention score
Thompson et al.[234]	2022	Brain White Matter Development Over the First 13 Years in Very Preterm and Typically Developing Children Based on the T (1)-w/T (2)-w Ratio	NEUROLOGY	age group too young/too old
Tideman et al.[235]	2000	Longitudinal follow-up of children born preterm: cognitive development at age 19	EARLY HUMAN DEVELOPMENT	age group too young/too old
Toijonen et al.[236]	2022	Impact of fetal presentation on neurodevelopmental outcome in a trial of preterm vaginal delivery: a nationwide, population-based record linkage study	ARCHIVES OF GYNECOLOGY AND OBSTETRICS	no sustained/selective attention score
Tommiska et al.[237]	2020	Analysis of neurodevelopmental outcomes of preadolescents born with extremely low weight revealed impairments in multiple developmental domains despite absence of cognitive impairment	HEALTH SCIENCE REPORTS	no sustained/selective attention score
Torrioli et al.[238]	2000	Perceptual-motor, visual and cognitive ability in very low birthweight preschool children without neonatal ultrasound abnormalities	BRAIN & DEVELOPMENT	focused on birth weight, not gestational age
Trickett et al.[239]	2022	Neuropsychological abilities underpinning academic attainment in children born extremely preterm	CHILD NEUROPSYCHOLOGY	age group too young/too old
Tseng et al.[240]	2019	Working Memory Training Is Associated with Changes in Resting State Functional Connectivity in Children Who Were Born Extremely Preterm: a Randomized Controlled Trial	JOURNAL OF COGNITIVE ENHANCEMENT	no sustained/selective attention score

Twilhaar et al.[241]	2020	Voluntary and Involuntary Control of Attention in Adolescents Born Very Preterm: A Study of Eye Movements	CHILD DEVELOPMENT	age group too young/too old
Twilhaar et al.[242]	2020	Neurocognitive processes underlying academic difficulties in very preterm born adolescents	CHILD NEUROPSYCHOLOGY	age group too young/too old
Twilhaar et al.[243]	2018	A randomised trial of enteral glutamine supplementation for very preterm children showed no beneficial or adverse long-term neurodevelopmental outcomes	ACTA PAEDIATRICA	no term born control group
Urben et al.[244]	2017	Gestational age and gender influence on executive control and its related neural structures in preterm-born children at 6 years of age	CHILD NEUROPSYCHOLOGY	no control group
van Baar et al.[245]	2020	Reliability and Validity of the Utrecht Tasks for Attention in Toddlers Using Eye Tracking (UTATE)	FRONTIERS IN PSYCHOLOGY	feasibility study
van der Burg et al.[246]	2017	Maternal obesity and attention-related symptoms in the preterm offspring	EARLY HUMAN DEVELOPMENT	ADHD
van der Ree et al.[247]	2011	Functional impairments at school age of preterm born children with late-onset sepsis	EARLY HUMAN DEVELOPMENT	no control group
van Gils et al.[248]	2020	Brain Damage and Visuospatial Impairments: Exploring Early Structure-Function Associations in Children Born Very Preterm	PEDIATRIC NEUROLOGY	no sustained/selective attention score
van Houdt et al.[249]	2019	Effects of Executive Function Training on Attentional, Behavioral and Emotional Functioning and Self-Perceived Competence in Very Preterm Children: A Randomized Controlled Trial	FRONTIERS IN PSYCHOLOGY	no control group
van Houdt et al.[250]	2021	Executive function training in very preterm children: a randomized controlled trial	EUROPEAN CHILD & ADOLESCENT PSYCHIATRY	no control group
van Houdt et al.[251]	2019	Developmental outcomes of very preterm children with high parental education level	EARLY HUMAN DEVELOPMENT	no sustained/selective attention score
van Houdt et al.[252]	2020	Subtypes of behavioral functioning in 8-12 year old very preterm children	EARLY HUMAN DEVELOPMENT	no sustained/selective attention score
Van Hus et al.[253]	2014	Motor impairment in very preterm-born children: links with other developmental deficits at 5 years of age	DEVELOPMENTAL MEDICINE AND CHILD NEUROLOGY	no sustained/selective attention score
Vanderbilt et al.[254]	2011	Mental Health Concerns of the Premature Infant Through the Lifespan	PEDIATRIC CLINICS OF NORTH AMERICA	no sustained/selective attention score
Vederhus et al.[255]	2010	Health related quality of life after extremely preterm birth: a matched controlled cohort study	HEALTH AND QUALITY OF LIFE OUTCOMES	no sustained/selective attention score
Verkerk et al.[256]	2016	Attention in 3-Year-Old Children with VLBW and Relationships with Early School Outcomes	PHYSICAL AND OCCUPATIONAL THERAPY IN PEDIATRICS	focused on birth weight, not gestational age

Walczak-Kozlowska et al.[257]	2022	Heterogeneity of the attentional system's efficiency among very prematurely born pre-schoolers	CHILD NEUROPSYCHOLOGY	multiple publication of an included dataset
Walczak-Kozlowska et al.[258]	2020	Attentional System of Very Prematurely Born Preschoolers	DEVELOPMENTAL PSYCHOLOGY	no sustained/selective attention score
Wehrle et al.[259]	2018	Altered resting-state functional connectivity in children and adolescents born very preterm short title	NEUROIMAGE-CLINICAL	no sustained/selective attention score
Welch et al.[260]	2015	Family Nurture Intervention in the Neonatal Intensive Care Unit improves social-relatedness, attention, and neurodevelopment of preterm infants at 18 months in a randomized controlled trial	JOURNAL OF CHILD PSYCHOLOGY AND PSYCHIATRY	no sustained/selective attention score
Wheelock et al.[261]	2021	Functional Connectivity Network Disruption Underlies Domain-Specific Impairments in Attention for Children Born Very Preterm	CEREBRAL CORTEX	age group too young/too old
Wheelock et al.[262]	2018	Altered functional network connectivity relates to motor development in children born very preterm	NEUROIMAGE	no sustained/selective attention score
Wilson-Ching et al.[263]	2013	Attention Difficulties in a Contemporary Geographic Cohort of Adolescents Born Extremely Preterm/Extremely Low Birth Weight	JOURNAL OF THE INTERNATIONAL NEUROPSYCHOLOGICAL SOCIETY	ADHD
Wong et al.[264]	2014	Kindergarten classroom functioning of extremely preterm/extremely low birth weight children	EARLY HUMAN DEVELOPMENT	no sustained/selective attention score
Woodward et al.[265]	2011	Neonatal White Matter Abnormalities Predict Global Executive Function Impairment in Children Born Very Preterm	DEVELOPMENTAL NEUROPSYCHOLOGY	age group too young/too old
Yoneda et al.[266]	2021	Pre-eclampsia Complicated With Maternal Renal Dysfunction Is Associated With Poor Neurological Development at 3 Years Old in Children Born Before 34 Weeks of Gestation	FRONTIERS IN PEDIATRICS	no sustained/selective attention score
You et al.[267]	2019	A study on the neurodevelopment outcomes of late preterm infants	BMC NEUROLOGY	no sustained/selective attention score
Yu et al.[268]	2020	Behavioural Problems Amongst Pre-School Children in Chongqing, China: Current Situation and Influencing Factors	RISK MANAGEMENT AND HEALTHCARE POLICY	no sustained/selective attention score
Zhang et al.[269]	2024	Complete rupture of the pregnant uterus: A 12-year retrospective study	INTERNATIONAL JOURNAL OF GYNECOLOGY & OBSTETRICS	no sustained/selective attention score
Zivan et al.[270]	2021	Hyper-Reactivity to Salience Limits Social Interaction Among Infants Born Pre-term and Infant Siblings of Children With ASD	FRONTIERS IN PSYCHIATRY	autism
Zuccarini et al.[271]	2016	Object engagement and manipulation in extremely preterm and full term infants at 6 months of age	RESEARCH IN DEVELOPMENTAL DISABILITIES	no sustained/selective attention score

References

1. Aarnoudse-Moens, C.S.H.; Weisglas-Kuperus, N.; Duivenvoorden, H.J.; Goudoever, J.B. van; Oosterlaan, J. Executive Function and IQ Predict Mathematical and Attention Problems in Very Preterm Children. *PLOS ONE* **2013**, *8*, e55994, doi:10.1371/journal.pone.0055994.
2. Aarnoudse-Moens, C.S.H.; Twilhaar, E.S.; Oosterlaan, J.; van Veen, H.G.; Prins, P.J.M.; van Kaam, A.H.L.C.; van Wassenaer-Leemhuis, A.G. Executive Function Computerized Training in Very Preterm-Born Children: A Pilot Study. *Games for Health Journal* **2018**, *7*, 175–181, doi:10.1089/g4h.2017.0038.
3. Abernethy, L.; Cooke, R.; Foulder-Hughes, L. Caudate and Hippocampal Volumes, Intelligence, and Motor Impairment in 7-Year-Old Children Who Were Born Preterm. *PEDIATRIC RESEARCH* **2004**, *55*, 884–893, doi:10.1203/01.PDR.0000117843.21534.49.
4. Agarwal, P.K.; Zheng, Q.; Yang, P.H.; Shi, L.; Rajadurai, V.S.; Khoo, P.C.; Quek, B.H.; Daniel, L.M. Academic School Readiness in Children Born Very Preterm and Associated Risk Factors. *EARLY HUMAN DEVELOPMENT* **2021**, *155*, doi:10.1016/j.earlhumdev.2021.105325.
5. Alonso-Lopez, P.; Arroyas, M.; Beato, M.; Ruiz-Gonzalez, S.; Olabarrieta, I.; Garcia-Garcia, M.L. Respiratory, Cardio-Metabolic and Neurodevelopmental Long-Term Outcomes of Moderate to Late Preterm Birth: Not Just a near Term-Population. A Follow-up Study. *FRONTIERS IN MEDICINE* **2024**, *11*, doi:10.3389/fmed.2024.1381118.
6. Anderson, P.J.; Lee, K.J.; Roberts, G.; Spencer-Smith, M.M.; Thompson, D.K.; Seal, M.L.; Nosarti, C.; Grehan, A.; Josev, E.K.; Gathercole, S.; et al. Long-Term Academic Functioning Following Cogmed Working Memory Training for Children Born Extremely Preterm: A Randomized Controlled Trial. *JOURNAL OF PEDIATRICS* **2018**, *202*, 92-+, doi:10.1016/j.jpeds.2018.07.003.
7. Anderson, P.; Doyle, L.W.; Victorian Infant Collaborative Study Group Neurobehavioral Outcomes of School-Age Children Born Extremely Low Birth Weight or Very Preterm in the 1990s. *JAMA* **2003**, *289*, 3264–3272, doi:10.1001/jama.289.24.3264.
8. Andersson, A.K.; Almqvist, L.; Strand Brodd, K.; Harder, M. Meaningful Everyday Life Situations from the Perspective of Children Born Preterm: A Photo-Elicitation Interview Study with Six-Year-Old Children. *PLoS One* **2023**, *18*, e0284217, doi:10.1371/journal.pone.0284217.
9. Arhan, E.; Gücüyener, K.; Soysal, Ş.; Şalvarlı, Ş.; Gürses, M.A.; Serdaroğlu, A.; Demir, E.; Ergenekon, E.; Türkyılmaz, C.; Önal, E.; et al. Regional Brain Volume Reduction and Cognitive Outcomes in Preterm Children at Low Risk at 9 Years of Age. *Childs Nerv Syst* **2017**, *33*, 1317–1326, doi:10.1007/s00381-017-3421-2.
10. Ataman-Devrim, M.; Quigley, J.; Nixon, E. Preterm Toddlers' Joint Attention Characteristics during Dyadic Interactions with Their Mothers and Fathers Compared to Full-Term Toddlers at Age 2 Years. *INFANT BEHAVIOR & DEVELOPMENT* **2024**, *74*, doi:10.1016/j.infbeh.2023.101915.
11. Ataman-Devrim, M.; Nixon, E.; Quigley, J. Neonatal Risk and Coordinated Joint Attention Episodes with Mothers and Fathers Relate to Language Skills of Preterm Children Aged 2-4 Years. *COGNITIVE DEVELOPMENT* **2024**, *70*, doi:10.1016/j.cogdev.2024.101456.
12. Baron, I.S.; Erickson, K.; Ahronovich, M.D.; Baker, R.; Litman, F.R. Neuropsychological and Behavioral Outcomes of Extremely Low Birth Weight at Age Three. *DEVELOPMENTAL NEUROPSYCHOLOGY* **2011**, *36*, 5–21, doi:10.1080/87565641.2011.540526.
13. Baron, I.S.; Ahronovich, M.D.; Erickson, K.; Gidley Larson, J.C.; Litman, F.R. Age-Appropriate Early School Age Neurobehavioral Outcomes of Extremely Preterm Birth without Severe Intraventricular Hemorrhage: A Single Center Experience. *Early Human Development* **2009**, *85*, 191–196, doi:10.1016/j.earlhumdev.2008.09.411.
14. Baron, I.S.; Erickson, K.; Ahronovich, M.D.; Coulehan, K.; Baker, R.; Litman, F.R. Visuospatial and Verbal Fluency Relative Deficits in "complicated" Late-Preterm Preschool Children. *EARLY HUMAN DEVELOPMENT* **2009**, *85*, 751–754, doi:10.1016/j.earlhumdev.2009.10.002.
15. Beunders, V.A.A.; Vermeulen, M.J.; Roelants, J.A.; Rietema, N.; Swarte, R.M.C.; Reiss, I.K.M.; Pel, J.J.M.; Joosten, K.F.M.; Kooiker, M.J.G. Early Visuospatial Attention and Processing and Related Neurodevelopmental Outcome at 2 Years in Children Born Very Preterm. *Pediatr Res* **2021**, *90*, 608–616, doi:10.1038/s41390-020-01206-7.

16. Bijlsma, A.; van Gils, M.M.; Beunders, V.A.A.; Reiss, I.K.M.; Joosten, K.F.M.; Pel, J.J.M.; Kooiker, M.J.G.; Vermeulen, M.J. Visual Attention and Processing Function in Relation to Executive Functioning in Very Preterm-Born Children Aged 3 Years: A Prospective Cohort Study. *EUROPEAN JOURNAL OF PEDIATRICS* **2024**, *183*, 4519–4529, doi:10.1007/s00431-024-05720-2.
17. Bogicevic, L.; Verhoeven, M.; van Baar, A.L. Exploring Predictors at Toddler Age of Distinct Profiles of Attentional Functioning in 6-Year-Old Children Born Moderate-to-Late Preterm and Full Term. *PLOS ONE* **2021**, *16*, doi:10.1371/journal.pone.0254797.
18. Bogicevic, L.; Pascoe, L.; Nguyen, T.-N.-N.; Burnett, A.C.; Verhoeven, M.; Thompson, D.K.; Cheong, J.L.Y.; Inder, T.E.; van Baar, A.L.; Doyle, L.W.; et al. Individual Attention Patterns in Children Born Very Preterm and Full Term at 7 and 13 Years of Age. *Journal of the International Neuropsychological Society : JINS* **2021**, *27*, 970–980, doi:10.1017/S1355617720001411.
19. Bogičević, L.; Verhoeven, M.; Baar, A.L. van Toddler Skills Predict Moderate-to-Late Preterm Born Children’s Cognition and Behaviour at 6 Years of Age. *PLOS ONE* **2019**, *14*, e0223690, doi:10.1371/journal.pone.0223690.
20. Bogičević, L.; Verhoeven, M.; van Baar, A.L. Distinct Profiles of Attention in Children Born Moderate-to-Late Preterm at 6 Years. *Journal of Pediatric Psychology* **2020**, *45*, 685–694, doi:10.1093/jpepsy/jsaa038.
21. Bolk, J.; Farooqi, A.; Hafstrom, M.; Aden, U.; Serenius, F. Developmental Coordination Disorder and Its Association With Developmental Comorbidities at 6.5 Years in Apparently Healthy Children Born Extremely Preterm. *JAMA PEDIATRICS* **2018**, *172*, 765–774, doi:10.1001/jamapediatrics.2018.1394.
22. Bora, S.; Pritchard, V.E.; Chen, Z.; Inder, T.E.; Woodward, L.J. Neonatal Cerebral Morphometry and Later Risk of Persistent Inattention/Hyperactivity in Children Born Very Preterm. *JOURNAL OF CHILD PSYCHOLOGY AND PSYCHIATRY* **2014**, *55*, 828–838, doi:10.1111/jcpp.12200.
23. Botellero, V.L.; Skranes, J.; Bjuland, K.J.; Haberg, A.K.; Lydersen, S.; Brubakk, A.-M.; Indredavik, M.S.; Martinussen, M. A Longitudinal Study of Associations between Psychiatric Symptoms and Disorders and Cerebral Gray Matter Volumes in Adolescents Born Very Preterm. *BMC PEDIATRICS* **2017**, *17*, doi:10.1186/s12887-017-0793-0.
24. Breeman, L.D.; Jaekel, J.; Baumann, N.; Bartmann, P.; Baeuml, J.G.; Avram, M.; Sorg, C.; Wolke, D. Infant Regulatory Problems, Parenting Quality and Childhood Attention Problems. *EARLY HUMAN DEVELOPMENT* **2018**, *124*, 11–16, doi:10.1016/j.earlhumdev.2018.07.009.
25. Breeman, L.D.; Jaekel, J.; Baumann, N.; Bartmann, P.; Wolke, D. Attention Problems in Very Preterm Children from Childhood to Adulthood: The Bavarian Longitudinal Study. *J Child Psychol Psychiatry* **2016**, *57*, 132–140, doi:10.1111/jcpp.12456.
26. Brogan, E.; Cragg, L.; Gilmore, C.; Marlow, N.; Simms, V.; Johnson, S. Inattention in Very Preterm Children: Implications for Screening and Detection. *Arch Dis Child* **2014**, *99*, 834–839, doi:10.1136/archdischild-2013-305532.
27. Brown, R.N.; Pascoe, L.; Treyvaud, K.; McMahon, G.; Nguyen, T.-N.-N.; Ellis, R.; Stedall, P.; Haebich, K.; Collins, S.E.; Cheong, J.; et al. Early Parenting Behaviour Is Associated with Complex Attention Outcomes in Middle to Late Childhood in Children Born Very Preterm. *Child Neuropsychology* **2023**, *29*, 165–182, doi:10.1080/09297049.2022.2075334.
28. Brown, R.N.; Burnett, A.C.; Thompson, D.K.; Spittle, A.J.; Ellis, R.; Cheong, J.L.Y.; Doyle, L.W.; Pascoe, L.; Anderson, P.J. Motor Performance and Attention Outcomes in Children Born Very Preterm. *DEVELOPMENTAL MEDICINE AND CHILD NEUROLOGY* **2023**, doi:10.1111/dmcn.15620.
29. Bucci, M.P.; Caldani, S.; Boutillier, B.; Frérot, A.; Farnoux, C.; Virlouvet, A.-L.; Rideau-Batista-Novais, A.; Trousson, C.; Biran, V. Immature Brain Structures Were Associated with Poorer Eye Movement Performance at 8 Years of Age in Preterm Born Children. *Acta Paediatr* **2022**, *111*, 559–565, doi:10.1111/apa.16197.
30. Bul, K.C.M.; van Baar, A.L. Behavior Problems in Relation to Sustained Selective Attention Skills of Moderately Preterm Children. *J Dev Phys Disabil* **2012**, *24*, 111–123, doi:10.1007/s10882-011-9258-9.
31. Burnett, A.C.; Youssef, G.; Anderson, P.J.; Duff, J.; Doyle, L.W.; Cheong, J.L.Y.; Callanan, C.; Carse, E.; Charlton, M.P.; Davis, N.; et al. Exploring the “Preterm Behavioral Phenotype” in

Children Born Extremely Preterm. *JOURNAL OF DEVELOPMENTAL AND BEHAVIORAL PEDIATRICS* **2019**, *40*, 200–207, doi:10.1097/DBP.0000000000000646.
32. Butcher, P.R.; van Braeckel, K.; Bouma, A.; Einspieler, C.; Stremmelaar, E.F.; Bos, A.F. The Quality of Preterm Infants' Spontaneous Movements: An Early Indicator of Intelligence and Behaviour at School Age. *JOURNAL OF CHILD PSYCHOLOGY AND PSYCHIATRY* **2009**, *50*, 920–930, doi:10.1111/j.1469-7610.2009.02066.x.
33. Cainelli, E.; Vedovelli, L.; Wigley, I.L.C.M.; Bisiacchi, P.S.; Suppiej, A. Neonatal Spectral EEG Is Prognostic of Cognitive Abilities at School Age in Premature Infants without Overt Brain Damage. *EUROPEAN JOURNAL OF PEDIATRICS* **2021**, *180*, 909–918, doi:10.1007/s00431-020-03818-x.
34. Camerota, M.; Castellanos, F.X.; Carter, B.S.; Check, J.; Helderman, J.; Hofheimer, J.A.; McGowan, E.C.; Neal, C.R.; Pastyrnak, S.L.; Smith, L.M.; et al. Trajectories of Attention Problems in Preschoolers Born Very Preterm. *J Child Psychol Psychiatry* **2025**, *66*, 667–676, doi:10.1111/jcpp.14074.
35. Camerota, M.; Lester, B.M.; Castellanos, F.X.; Carter, B.S.; Check, J.; Helderman, J.; Hofheimer, J.A.; McGowan, E.C.; Neal, C.R.; Pastyrnak, S.L.; et al. Epigenome-Wide Association Study Identifies Neonatal DNA Methylation Associated with Two-Year Attention Problems in Children Born Very Preterm. *Transl Psychiatry* **2024**, *14*, 126, doi:10.1038/s41398-024-02841-y.
36. Campbell, C.; Horlin, C.; Reid, C.; McMichael, J.; Forrest, L.; Brydges, C.; French, N.; Anderson, M. How Do You Think She Feels? Vulnerability in Empathy and the Role of Attention in School-Aged Children Born Extremely Preterm. *BRITISH JOURNAL OF DEVELOPMENTAL PSYCHOLOGY* **2015**, *33*, 312–323, doi:10.1111/bjdp.12091.
37. Caravale, B.; Tozzi, C.; Albino, G.; Vicari, S. Cognitive Development in Low Risk Preterm Infants at 3-4 Years of Life. *ARCHIVES OF DISEASE IN CHILDHOOD-FETAL AND NEONATAL EDITION* **2005**, *90*, F474–F479, doi:10.1136/adc.2004.070284.
38. Caravale, B.; Sette, S.; Cannoni, E.; Marano, A.; Riolo, E.; Devescovi, A.; De Curtis, M.; Bruni, O. Sleep Characteristics and Temperament in Preterm Children at Two Years of Age. *JOURNAL OF CLINICAL SLEEP MEDICINE* **2017**, *13*, 1081–1088, doi:10.5664/jcsm.6728.
39. Caravale, B.; Mirante, N.; Vagnoni, C.; Vicari, S. Change in Cognitive Abilities over Time during Preschool Age in Low Risk Preterm Children. *EARLY HUMAN DEVELOPMENT* **2012**, *88*, 363–367, doi:10.1016/j.earlhumdev.2011.09.011.
40. Cherkes-Julkowski, M. Learning Disability, Attention-Deficit Disorder, and Language Impairment as Outcomes of Prematurity: A Longitudinal Descriptive Study. *JOURNAL OF LEARNING DISABILITIES* **1998**, *31*, 294–306, doi:10.1177/002221949803100309.
41. Chin, W.-C.; Wu, W.-C.; Hsu, J.-F.; Tang, I.; Yao, T.-C.; Huang, Y.-S. Correlation Analysis of Attention and Intelligence of Preterm Infants at Preschool Age: A Premature Cohort Study. *International Journal of Environmental Research and Public Health* **2023**, *20*, 3357, doi:10.3390/ijerph20043357.
42. COHEN, S.; PARMELEE, A. PREDICTION OF 5-YEAR STANFORD-BINET SCORES IN PRETERM INFANTS. *CHILD DEVELOPMENT* **1983**, *54*, 1242–1253.
43. Coratti, G.; Mallardi, M.; Pede, E.; Mangano, G.; Sicolo, A.; D'Argenzio, M.; Gallini, F.; Romeo, D.M.; Chieffo, D.; Vento, G.; et al. Assessment of Early Attention in an Italian Cohort of Preschooler Preterm Children Using the Early Childhood Attention Battery. *EUROPEAN JOURNAL OF PEDIATRICS* **2024**, *184*, doi:10.1007/s00431-024-05832-9.
44. Cosentino-Rocha, L.; Klein, V.C.; Martins Linhares, M.B. Effects of Preterm Birth and Gender on Temperament and Behavior in Children. *INFANT BEHAVIOR & DEVELOPMENT* **2014**, *37*, 446–456, doi:10.1016/j.infbeh.2014.04.003.
45. Crockett, L.K.; Brownell, M.D.; Heaman, M.I.; Ruth, C.A.; Prior, H.J. Examining Early Childhood Health Outcomes of Children Born Late Preterm in Urban Manitoba. *MATERNAL AND CHILD HEALTH JOURNAL* **2017**, *21*, 2141–2148, doi:10.1007/s10995-017-2329-5.
46. Crowther, C.A.; Doyle, L.W.; Haslam, R.R.; Hiller, J.E.; Harding, J.E.; Robinson, J.S.; ACTORDS Study Grp Outcomes at 2 Years of Age after Repeat Doses of Antenatal Corticosteroids. *NEW ENGLAND JOURNAL OF MEDICINE* **2007**, *357*, 1179–1189, doi:10.1056/NEJMoa071152.

47. Cserjesi, R.; Van Braeckel, K.N.; Timmerman, M.; Butcher, P.R.; Kerstjens, J.M.; Reijneveld, S.A.; Bouma, A.; Bos, A.F.; Geuze, R.H. Patterns of Functioning and Predictive Factors in Children Born Moderately Preterm or at Term. *DEVELOPMENTAL MEDICINE AND CHILD NEUROLOGY* **2012**, *54*, 710–715, doi:10.1111/j.1469-8749.2012.04328.x.
48. Cusin Lamonica, D.A.; Becaro, C.K.; Borba, A.C.; Maximino, L.D.P.; Aceituno da Costa, A.R.; Ribeiro, C.D.C. Communicative Performance and Vocabulary Domain in Preschool Preterm Infants. *JOURNAL OF APPLIED ORAL SCIENCE* **2018**, *26*, doi:10.1590/1678-7757-2017-0186.
49. Dai, D.W.T.; Wouldes, T.A.; Brown, G.T.L.; Tottman, A.C.; Alsweiler, J.M.; Gamble, G.D.; Harding, J.E.; Piano Study Grp Relationships between Intelligence, Executive Function and Academic Achievement in Children Born Very Preterm. *EARLY HUMAN DEVELOPMENT* **2020**, *148*, doi:10.1016/j.earlhumdev.2020.105122.
50. Danks, M.; Cherry, K.; Burns, Y.R.; Gray, P.H. Are Behaviour Problems in Extremely Low-Birthweight Children Related to Their Motor Ability? *ACTA PAEDIATRICA* **2017**, *106*, 568–572, doi:10.1111/apa.13712.
51. Datin-Dorriere, V.; Borst, G.; Guillois, B.; Cachia, A.; Poirel, N. The Forest, the Trees, and the Leaves in Preterm Children: The Impact of Prematurity on a Visual Search Task Containing Three-Level Hierarchical Stimuli. *EUROPEAN CHILD & ADOLESCENT PSYCHIATRY* **2021**, *30*, 253–260, doi:10.1007/s00787-020-01510-x.
52. de Jong, M.; Verhoeven, M.; van Baar, A.L. Attention Capacities of Preterm and Term Born Toddlers: A Multi-Method Approach. *Early Human Development* **2015**, *91*, 761–768, doi:10.1016/j.earlhumdev.2015.08.015.
53. de Kieviet, J.F.; Heslenfeld, D.J.; Pouwels, P.J.W.; Lafeber, H.N.; Vermeulen, R.J.; van Elburg, R.M.; Oosterlaan, J. A Crucial Role for White Matter Alterations in Interference Control Problems of Very Preterm Children. *Pediatr Res* **2014**, *75*, 731–737, doi:10.1038/pr.2014.31.
54. de Kieviet, J.F.; van Elburg, R.M.; Lafeber, H.N.; Oosterlaan, J. Attention Problems of Very Preterm Children Compared with Age-Matched Term Controls at School-Age. *J Pediatr* **2012**, *161*, 824–829, doi:10.1016/j.jpeds.2012.05.010.
55. De Schuymer, L.; De Groote, I.; Desoete, A.; Roeyers, H. Gaze Aversion during Social Interaction in Preterm Infants: A Function of Attention Skills? *Infant Behav Dev* **2012**, *35*, 129–139, doi:10.1016/j.infbeh.2011.08.002.
56. Dean, B.; O'Carroll, S.; Ginnell, L.; Ledsham, V.; Telford, E.; Sparrow, S.; Boardman, J.P.; Fletcher-Watson, S. Longitudinal Assessment of Social Cognition in Infants Born Preterm Using Eye-Tracking and Parent-Child Play. *INFANT AND CHILD DEVELOPMENT* **2021**, *30*, doi:10.1002/icd.2275.
57. Della Rosa, P.A.; Canini, M.; Marchetta, E.; Cirillo, S.; Pontesilli, S.; Scotti, R.; Sora, M.G.N.; Poloniato, A.; Barera, G.; Falini, A.; et al. The Effects of the Functional Interplay between the Default Mode and Executive Control Resting State Networks on Cognitive Outcome in Preterm Born Infants at 6 Months of Age. *BRAIN AND COGNITION* **2021**, *147*, doi:10.1016/j.bandc.2020.105669.
58. Delobel-Ayoub, M.; Arnaud, C.; White-Koning, M.; Casper, C.; Pierrat, V.; Garel, M.; Burguet, A.; Roze, J.-C.; Matis, J.; Picaud, J.-C.; et al. Behavioral Problems and Cognitive Performance at 5 Years of Age After Very Preterm Birth: The EPIPAGE Study. *PEDIATRICS* **2009**, *123*, 1485–1492, doi:10.1542/peds.2008-1216.
59. den Heijer, A.E.; Jansen, A.S.N.; van Kersbergen, M.; van Dokkum, N.H.; Reijneveld, S.A.; Spikman, J.M.; de Kroon, M.L.A.; Bos, A.F. Neurocognitive Outcomes in Moderately Preterm Born Adolescents. *EARLY HUMAN DEVELOPMENT* **2024**, *193*, doi:10.1016/j.earlhumdev.2024.106020.
60. Dilworth-Bart, J.; Poehlmann, J.; Hilgendorf, A.E.; Miller, K.; Lambert, H. Maternal Scaffolding and Preterm Toddlers' Visual-Spatial Processing and Emerging Working Memory. *JOURNAL OF PEDIATRIC PSYCHOLOGY* **2010**, *35*, 209–220, doi:10.1093/jpepsy/jsp048.
61. Espírito Santo, J.L. do; Portuguez, M.W.; Nunes, M.L. Cognitive and Behavioral Status of Low Birth Weight Preterm Children Raised in a Developing Country at Preschool Age. *J Pediatr (Rio J)* **2009**, *85*, 35–41, doi:10.2223/JPED.1859.

62. Domellof, E.; Johansson, A.-M.; Farooqi, A.; Domellof, M.; Ronnqvist, L. Risk for Behavioral Problems Independent of Cognitive Functioning in Children Born at Low Gestational Ages. *FRONTIERS IN PEDIATRICS* **2020**, *8*, doi:10.3389/fped.2020.00311.
63. Dotinga, B.M.; Eshuis, M.S.; Bocca-Tjeertes, I.F.; Kerstjens, J.M.; Van Braeckel, K.N.J.A.; Reijneveld, S.A.; Bos, A.F. Longitudinal Growth and Neuropsychological Functioning at Age 7 in Moderate and Late Preterms. *Pediatrics* **2016**, *138*, e20153638, doi:10.1542/peds.2015-3638.
64. Downes, M.; Kelly, D.; Day, K.; Marlow, N.; de Haan, M. Visual Attention Control Differences in 12-Month-Old Preterm Infants. *Infant Behavior and Development* **2018**, *50*, 180–188, doi:10.1016/j.infbeh.2018.01.002.
65. Downey, L.C.; O'Shea, T.M.; Allred, E.N.; Kuban, K.; McElrath, T.F.; Warner, D.D.; Ware, J.; Hecht, J.L.; Onderdonk, A.; Leviton, A.; et al. Antenatal and Early Postnatal Antecedents of Parent-Reported Attention Problems at 2 Years of Age. *JOURNAL OF PEDIATRICS* **2015**, *166*, 20-U270, doi:10.1016/j.jpeds.2014.08.004.
66. Doyle, L.W.; Anderson, P.J.; Haslam, R.; Lee, K.J.; Crowther, C.; Australasian Collaborative Trial School-Age Outcomes of Very Preterm Infants After Antenatal Treatment With Magnesium Sulfate vs Placebo. *JAMA-JOURNAL OF THE AMERICAN MEDICAL ASSOCIATION* **2014**, *312*, 1105–1113, doi:10.1001/jama.2014.11189.
67. Dueker, G.; Chen, J.; Cowling, C.; Haskin, B. Early Developmental Outcomes Predicted by Gestational Age From 35 to 41 Weeks. *OBSTETRICAL & GYNECOLOGICAL SURVEY* **2017**, *72*, 211–212, doi:10.1097/01.ogx.0000515491.34643.5f.
68. Dupin, R.; Laurent, J.-P.; Stauder, J.E.A.; Saliba, E. Auditory Attention Processing in 5-Year-Old Children Born Preterm: Evidence from Event-Related Potentials. *Developmental Medicine & Child Neurology* **2000**, *42*, 476–480, doi:10.1111/j.1469-8749.2000.tb00351.x.
69. Einaudi, M.-A.; Busuttil, M.; Monnier, A.-S.; Chanus, I.; Palix, C.; Gire, C. Neuropsychological Screening of a Group of Preterm Twins: Comparison with Singletons. *CHILDS NERVOUS SYSTEM* **2008**, *24*, 225–230, doi:10.1007/s00381-007-0422-6.
70. El-Mazahi, M.; El-Mahdi, M.; El-Khaleeg, H.; Gomaa, G. Cognitive and Behavioural Outcome of Preterm Versus Full Term Infants, at School Entry Age. *Trends in Medical Research* **2014**, *9*, 44–52, doi:10.3923/tmr.2014.44.52.
71. Ernstad, E.G.; Hanson, C.; Wanggren, K.; Thurin-Kjellberg, A.; Soderberg, C.H.; Lundberg, E.S.; Petzold, M.; Wennerholm, U.-B.; Bergh, C. Preimplantation Genetic Testing and Child Health: A National Register-Based Study. *HUMAN REPRODUCTION* **2023**, *38*, 739–750, doi:10.1093/humrep/dead021.
72. Eryigit-Madzwamuse, S.; Wolke, D. Attention Problems in Relation to Gestational Age at Birth and Smallness for Gestational Age. *Early Hum Dev* **2015**, *91*, 131–138, doi:10.1016/j.earlhumdev.2015.01.004.
73. Espy, K.A.; Senn, T.E.; Charak, D.A.; Tyler, J.; Wiebe, S.A. Perinatal pH and Neuropsychological Outcomes at Age 3 Years in Children Born Preterm: An Exploratory Study. *Developmental Neuropsychology* **2007**, *32*, 669–682, doi:10.1080/87565640701376003.
74. Espy, K.; Stalets, M.; McDiarmid, M.; Senn, T.; Cwik, M.; Hamby, A. Executive Functions in Preschool Children Born Preterm: Application of Cognitive Neuroscience Paradigms. *CHILD NEUROPSYCHOLOGY* **2002**, *8*, 83–92, doi:10.1076/chin.8.2.83.8723.
75. Fan, R.G.; Portuguez, M.W.; Nunes, M.L. Cognition, Behavior and Social Competence of Preterm Low Birth Weight Children at School Age. *CLINICS* **2013**, *68*, 915–921, doi:10.6061/clinics/2013(07)05.
76. Farooqi, A.; Hägglöf, B.; Serenius, F. Behaviours Related to Executive Functions and Learning Skills at 11 Years of Age after Extremely Preterm Birth: A Swedish National Prospective Follow-up Study. *Acta Paediatr* **2013**, *102*, 625–634, doi:10.1111/apa.12219.
77. Filippa, M.; Lordier, L.; Lejeune, F.; De Almeida, J.S.; Huppi, P.S.; Barcos-Munoz, F.; Monaci, M.G.; Borradori-Tolsa, C. Effect of an Early Music Intervention on Emotional and Neurodevelopmental Outcomes of Preterm Infants at 12 and 24 Months. *FRONTIERS IN PSYCHOLOGY* **2024**, *15*, doi:10.3389/fpsyg.2024.1443080.

78. Fjørtoft, T.; Grunewaldt, K.H.; Løhaugen, G.C.C.; Mørkved, S.; Skranes, J.; Evensen, K.A.I. Adaptive Behavior in 10-11 Year Old Children Born Preterm with a Very Low Birth Weight (VLBW). *Eur J Paediatr Neurol* **2015**, *19*, 162–169, doi:10.1016/j.ejpn.2014.11.006.
79. Foulder-Hughes, L.A.; Cooke, R.W.I. Motor, Cognitive, and Behavioural Disorders in Children Born Very Preterm. *Dev Med Child Neurol* **2003**, *45*, 97–103.
80. Frye, R.E.; Hasan, K.; Malmberg, B.; Desouza, L.; Swank, P.; Smith, K.; Landry, S. Superior Longitudinal Fasciculus and Cognitive Dysfunction in Adolescents Born Preterm and at Term. *Dev Med Child Neurol* **2010**, *52*, 760–766, doi:10.1111/j.1469-8749.2010.03633.x.
81. Garcia-Bermudez, O.; Cruz-Quintana, F.; Perez-Garcia, M.; Hidalgo-Ruzzante, N.; Fernandez-Alcantara, M.; Nieves Perez-Marfil, M. Improvement of Executive Functions after the Application of a Neuropsychological Intervention Program (PEFEN) in Pre-Term Children. *CHILDREN AND YOUTH SERVICES REVIEW* **2019**, *98*, 328–336, doi:10.1016/j.childyouth.2018.10.035.
82. Geldof, C.J.A.; de Kieviet, J.F.; Dik, M.; Kok, J.H.; van Wassenaer-Leemhuis, A.G.; Oosterlaan, J. Visual Search and Attention in Five-Year-Old Very Preterm/Very Low Birth Weight Children. *EARLY HUMAN DEVELOPMENT* **2013**, *89*, 983–988, doi:10.1016/j.earlhumdev.2013.08.021.
83. Geva, R.; Yaron, H.; Kuint, J. Neonatal Sleep Predicts Attention Orienting and Distractibility. *J Atten Disord* **2016**, *20*, 138–150, doi:10.1177/1087054713491493.
84. Gillenson, C.J.; Bagner, D.M.; Darcy Mahoney, A.; Baralt, M. A Preliminary Study of Executive Functioning in Preterm-Born Children: A Bilingual Advantage. *Adv Neonatal Care* **2023**, *23*, E121–E128, doi:10.1097/ANC.0000000000001106.
85. Gire, C.; Beltran Anzola, A.; Marret, S.; Foix L'Hélias, L.; Roze, J.-C.; Granier, M.; Patural, H.; Lecomte, B.; Guillois, B.; Souksi Medioni, I.; et al. Cognitive Training for Visuospatial Processing in Children Aged 5½ to 6 Years Born Very Preterm With Working Memory Dysfunction: A Randomized Clinical Trial. *JAMA Network Open* **2023**, *6*, e2331988, doi:10.1001/jamanetworkopen.2023.31988.
86. Gire, C.; Resseguier, N.; Brevaut-Malaty, V.; Marret, S.; Cambonie, G.; Souksi-Medioni, I.; Mueller, J.-B.; Garcia, P.; Berbis, J.; Tosello, B.; et al. Quality of Life of Extremely Preterm School-Age Children without Major Handicap: A Cross-Sectional Observational Study. *ARCHIVES OF DISEASE IN CHILDHOOD* **2019**, *104*, 333-+, doi:10.1136/archdischild-2018-315046.
87. Gomaa, N.; Konwar, C.; Gladish, N.; Au-Young, S.H.; Guo, T.; Sheng, M.; Merrill, S.M.; Kelly, E.; Chau, V.; Branson, H.M.; et al. Association of Pediatric Buccal Epigenetic Age Acceleration With Adverse Neonatal Brain Growth and Neurodevelopmental Outcomes Among Children Born Very Preterm With a Neonatal Infection. *JAMA Netw Open* **2022**, *5*, e2239796, doi:10.1001/jamanetworkopen.2022.39796.
88. Gomot, M.; Bruneau, N.; Laurent, J.-P.; Barthelemy, C.; Saliba, E. Left Temporal Impairment of Auditory Information Processing in Prematurely Born 9-Year-Old Children:: An Electrophysiological Study. *INTERNATIONAL JOURNAL OF PSYCHOPHYSIOLOGY* **2007**, *64*, 123–129, doi:10.1016/j.ijpsycho.2007.01.003.
89. Gould, J.F.; Hunt, E.; Roberts, R.M.; Louise, J.; Collins, C.T.; Makrides, M. Can the Bayley Scales of Infant Development at 18 Months Predict Child Behaviour at 7 Years? *JOURNAL OF PAEDIATRICS AND CHILD HEALTH* **2019**, *55*, 74–81, doi:10.1111/jpc.14163.
90. Griffiths, S.T.; Gundersen, H.; Neto, E.; Elgen, I.; Markestad, T.; Aukland, S.M.; Hugdahl, K. fMRI: Blood Oxygen Level–Dependent Activation during a Working Memory–Selective Attention Task in Children Born Extremely Preterm. *Pediatr Res* **2013**, *74*, 196–205, doi:10.1038/pr.2013.79.
91. Griffiths, S.T.; Aukland, S.M.; Markestad, T.; Eide, G.E.; Elgen, I.; Craven, A.R.; Hugdahl, K. Association between Brain Activation (fMRI), Cognition and School Performance in Extremely Preterm and Term Born Children. *SCANDINAVIAN JOURNAL OF PSYCHOLOGY* **2014**, *55*, 427–432, doi:10.1111/sjop.12145.
92. Grunewaldt, K.H.; Skranes, J.; Brubakk, A.-M.; Lahaugen, G.C.C. Computerized Working Memory Training Has Positive Long-Term Effect in Very Low Birthweight Preschool Children. *DEVELOPMENTAL MEDICINE AND CHILD NEUROLOGY* **2016**, *58*, 195–201, doi:10.1111/dmcn.12841.

93. Grunewaldt, K.H.; Lohaugen, G.C.C.; Austeng, D.; Brubakk, A.-M.; Skranes, J. Working Memory Training Improves Cognitive Function in VLBW Preschoolers. *PEDIATRICS* **2013**, *131*, E747–E754, doi:10.1542/peds.2012-1965.
94. Grunewaldt, K.H.; Fjortoft, T.; Bjuland, K.J.; Brubakk, A.-M.; Eikenes, L.; Haberg, A.K.; Lohaugen, G.C.C.; Skranes, J. Follow-up at Age 10 Years in ELBW Children - Functional Outcome, Brain Morphology and Results from Motor Assessments in Infancy. *EARLY HUMAN DEVELOPMENT* **2014**, *90*, 571–578, doi:10.1016/j.earlhumdev.2014.07.005.
95. Hall, J.; Jaekel, J.; Wolke, D. Gender Distinctive Impacts of Prematurity and Small for Gestational Age (SGA) on Age-6 Attention Problems. *CHILD AND ADOLESCENT MENTAL HEALTH* **2012**, *17*, 238–245, doi:10.1111/j.1475-3588.2012.00649.x.
96. Hebert-Myers, H.; Guttentag, C.L.; Swank, P.R.; Smith, K.E.; Landry, S.H. The Importance of Language, Social, and Behavioral Skills Across Early and Later Childhood as Predictors of Social Competence With Peers. *Applied Developmental Science* **2006**, *10*, 174–187, doi:10.1207/s1532480xads1004_2.
97. Hellgren, K.; Halberda, J.; Forsman, L.; Aden, U.; Libertus, M. Compromised Approximate Number System Acuity in Extremely Preterm School-Aged Children. *DEVELOPMENTAL MEDICINE AND CHILD NEUROLOGY* **2013**, *55*, 1109–1114, doi:10.1111/dmcn.12206.
98. Hemgren, E.; Persson, K. Associations of Motor Co-Ordination and Attention with Motor-Perceptual Development in 3-Year-Old Preterm and Full-Term Children Who Needed Neonatal Intensive Care. *CHILD CARE HEALTH AND DEVELOPMENT* **2007**, *33*, 11–21, doi:10.1111/j.1365-2214.2006.00625.x.
99. HILLE, E.; DENOUDEN, A.; BAUER, L.; VANDENOUDENRIJN, C.; BRAND, R.; VERLOOVEVANHORICK, S. SCHOOL PERFORMANCE AT 9 YEARS OF AGE IN VERY PREMATURE AND VERY-LOW-BIRTH-WEIGHT INFANTS - PERINATAL RISK-FACTORS AND PREDICTORS AT 5 YEARS OF AGE. *JOURNAL OF PEDIATRICS* **1994**, *125*, 426–434, doi:10.1016/S0022-3476(05)83290-1.
100. Hodel, A.S.; Senich, K.L.; Jokinen, C.; Sasson, O.; Morris, A.R.; Thomas, K.M. Early Executive Function Differences in Infants Born Moderate-to-Late Preterm. *Early Hum Dev* **2017**, *113*, 23–30, doi:10.1016/j.earlhumdev.2017.07.007.
101. Hofheimer, J.A.; McGrath, M.; Musci, R.; Wu, G.; Polk, S.; Blackwell, C.K.; Stroustrup, A.; Annett, R.D.; Aschner, J.; Carter, B.S.; et al. Assessment of Psychosocial and Neonatal Risk Factors for Trajectories of Behavioral Dysregulation Among Young Children From 18 to 72 Months of Age. *JAMA NETWORK OPEN* **2023**, *6*, doi:10.1001/jamanetworkopen.2023.10059.
102. Imgrund, C.M.; Loeb, D.F.; Barlow, S.M. Expressive Language in Preschoolers Born Preterm: Results of Language Sample Analysis and Standardized Assessment. *JOURNAL OF SPEECH LANGUAGE AND HEARING RESEARCH* **2019**, *62*, 884–895, doi:10.1044/2018_JSLHR-L-18-0224.
103. Indredavik, M.S.; Vik, T.; Evensen, K.A.I.; Skranes, J.; Taraldsen, G.; Brubakk, A.-M. Perinatal Risk and Psychiatric Outcome in Adolescents Born Preterm With Very Low Birth Weight or Term Small for Gestational Age. *JOURNAL OF DEVELOPMENTAL AND BEHAVIORAL PEDIATRICS* **2010**, *31*, 286–294, doi:10.1097/DBP.0b013e3181d7b1d3.
104. Ionio, C.; Lista, G.; Veggiotti, P.; Colombo, C.; Ciuffo, G.; Daniele, I.; Landoni, M.; Scelsa, B.; Alfei, E.; Bova, S. Cognitive, Behavioral and Socioemotional Development in a Cohort of Preterm Infants at School Age: A Cross-Sectional Study. *Pediatric Reports* **2022**, *14*, 115–126, doi:10.3390/pediatric14010017.
105. Jaeger, D.A.; Gawehn, N.; Schölmerich, A.; Schneider, D.T.; Suchan, B. Reduction of the Event-Related Potential P3 in Preterm Born 5-Year-Old Healthy Children. *Clinical Neurophysiology* **2019**, *130*, 675–682, doi:10.1016/j.clinph.2019.02.003.
106. Jaekel, J.; Heuser, K.M.; Zapf, A.; Roll, C.; Nunez, F.B.; Bartmann, P.; Wolke, D.; Felderhoff-Mueser, U.; Huening, B. Preterm Children’s Long-Term Academic Performance after Adaptive Computerized Training: An Efficacy and Process Analysis of a Randomized Controlled Trial. *PEDIATRIC RESEARCH* **2021**, *89*, 1492–1499, doi:10.1038/s41390-020-01114-w.

107. Jaekel, J.; Wolke, D.; Bartmann, P. Poor Attention Rather than Hyperactivity/Impulsivity Predicts Academic Achievement in Very Preterm and Full-Term Adolescents. *Psychol Med* **2013**, *43*, 183–196, doi:10.1017/S0033291712001031.
108. Jaekel, J.; Eryigit-Madzwamuse, S.; Wolke, D. Preterm Toddlers' Inhibitory Control Abilities Predict Attention Regulation and Academic Achievement at Age 8 Years. *J Pediatr* **2016**, *169*, 87-92.e1, doi:10.1016/j.jpeds.2015.10.029.
109. Janson, E.; Koolschijn, P.C.M.P.; Schipper, L.; Boerma, T.D.; Wijnen, F.N.K.; de Boode, W.P.; van den Akker, C.H.P.; van der Stap, R.G.; Nuytemans, D.H.G.M.; Onland, W.; et al. Dolphin CONTINUE: A Multi-Center Randomized Controlled Trial to Assess the Effect of a Nutritional Intervention on Brain Development and Long-Term Outcome in Infants Born before 30 Weeks of Gestation. *BMC PEDIATRICS* **2024**, *24*, doi:10.1186/s12887-024-04849-1.
110. Jin, J.H.; Yoon, S.W.; Song, J.; Kim, S.W.; Chung, H.J. Long-Term Cognitive, Executive, and Behavioral Outcomes of Moderate and Late Preterm at School Age. *CLINICAL AND EXPERIMENTAL PEDIATRICS* **2020**, *63*, 219–225, doi:10.3345/kjp.2019.00647.
111. Johnson, S.; Wolke, D.; Hennessy, E.; Marlow, N. Educational Outcomes in Extremely Preterm Children: Neuropsychological Correlates and Predictors of Attainment. *DEVELOPMENTAL NEUROPSYCHOLOGY* **2011**, *36*, 74–95, doi:10.1080/87565641.2011.540541.
112. Joo, J.W.; Choi, J.Y.; Rha, D.; Kwak, E.H.; Park, E.S. Neuropsychological Outcomes of Preterm Birth in Children With No Major Neurodevelopmental Impairments in Early Life. *ANNALS OF REHABILITATION MEDICINE-ARM* **2015**, *39*, 676–685, doi:10.5535/arm.2015.39.5.676.
113. Joseph, R.M.; O'Shea, T.M.; Allred, E.N.; Heeren, T.; Hirtz, D.; Jara, H.; Leviton, A.; Kuban, K.C.K.; ELGAN Study Investigators Neurocognitive and Academic Outcomes at Age 10 Years of Extremely Preterm Newborns. *Pediatrics* **2016**, *137*, e20154343, doi:10.1542/peds.2015-4343.
114. Kallankari, H.; Kaukola, T.; Olsén, P.; Ojaniemi, M.; Hallman, M. Very Preterm Birth and Foetal Growth Restriction Are Associated with Specific Cognitive Deficits in Children Attending Mainstream School. *Acta Paediatr* **2015**, *104*, 84–90, doi:10.1111/apa.12811.
115. Kaul, Y.F.; Rosander, K.; von Hofsten, C.; Strand Brodd, K.; Holmstrom, G.; Hellstrom-Westas, L. Visual Tracking at 4 Months in Preterm Infants Predicts 6.5-Year Cognition and Attention. *PEDIATRIC RESEARCH* **2022**, *92*, 1082–1089, doi:10.1038/s41390-021-01895-8.
116. Kim, S.Y.; Kim, E.-K.; Song, H.; Cheon, J.-E.; Kim, B.N.; Kim, H.-S.; Shin, S.H. Association of Brain Microstructure and Functional Connectivity With Cognitive Outcomes and Postnatal Growth Among Early School-Aged Children Born With Extremely Low Birth Weight. *JAMA NETWORK OPEN* **2023**, *6*, doi:10.1001/jamanetworkopen.2023.0198.
117. Kim, E.S.; Kim, E.-K.; Kim, S.Y.; Song, I.G.; Jung, Y.H.; Shin, S.H.; Kim, H.-S.; Kim, J.I.; Kim, B.N.; Shin, M.-S. Cognitive and Behavioral Outcomes of School-Aged Children Born Extremely Preterm: A Korean Single-Center Study with Long-Term Follow-Up. *J Korean Med Sci* **2021**, *36*, e260, doi:10.3346/jkms.2021.36.e260.
118. Knops, N.B.B.; Sneeuw, K.C.A.; Brand, R.; Hille, E.T.M.; den Ouden, A.L.; Wit, J.-M.; Verloove-Vanhorick, S.P. Catch-up Growth up to Ten Years of Age in Children Born Very Preterm or with Very Low Birth Weight. *BMC pediatrics* **2005**, *5*, 26–26, doi:10.1186/1471-2431-5-26.
119. Kochukhova, O.; Kaul, Y.F.; Johansson, M.; Montgomery, C.; Holmstrom, G.; Brodd, K.S.; Hellstrom-Westas, L. Antenatal Steroids and Neurodevelopment in 12-Year-Old Children Born Extremely Preterm. *ACTA PAEDIATRICA* **2022**, *111*, 314–322, doi:10.1111/apa.16140.
120. Kooiker, M.J.G.; Swarte, R.M.C.; Smit, L.S.; Reiss, I.K.M. Perinatal Risk Factors for Visuospatial Attention and Processing Dysfunctions at 1 year of Age in Children Born between 26 and 32 weeks. *Early Human Development* **2019**, *130*, 71–79, doi:10.1016/j.earlhumdev.2019.01.015.
121. Krägeloh-Mann, I.; Toft, P.; Lunding, J.; Andresen, J.; Pryds, O.; Lou, H. Brain Lesions in Preterms: Origin, Consequences and Compensation. *ACTA PAEDIATRICA* **1999**, *88*, 897–908.
122. Kuban, K.C.K.; O'Shea, M.; Allred, E.N.; Fichorova, R.N.; Heeren, T.; Paneth, N.; Hirtz, D.; Dammann, O.; Leviton, A.; ELGAN Study Investigators The Breadth and Type of Systemic Inflammation and the Risk of Adverse Neurological Outcomes in Extremely Low Gestation Newborns. *PEDIATRIC NEUROLOGY* **2015**, *52*, 42–48, doi:10.1016/j.pediatrneurol.2014.10.005.

123. Kurpershoek, T.; Potharst-Sirag, E.S.; Aarnoudse-Moens, C.S.H.; van Wassenaer-Leemhuis, A.G. Minor Neurological Dysfunction in Five Year Old Very Preterm Children Is Associated with Lower Processing Speed. *EARLY HUMAN DEVELOPMENT* **2016**, *103*, 55–60, doi:10.1016/j.earlhumdev.2016.07.002.
124. Lambe, M.; Hultman, C.; Torrang, A.; MacCabe, J.; Cnattingius, S. Maternal Smoking during Pregnancy and School Performance at Age 15. *EPIDEMIOLOGY* **2006**, *17*, 524–530, doi:10.1097/01.ede.0000231561.49208.be.
125. Landsem, I.P.; Handegård, B.H.; Ulvund, S.E.; Tunby, J.; Kaaresen, P.I.; Rønning, J.A. Does An Early Intervention Influence Behavioral Development Until Age 9 in Children Born Prematurely? *Child Development* **2015**, *86*, 1063–1079, doi:10.1111/cdev.12368.
126. Langkamp, D.; Brazy, J. Risk for Later School Problems in Preterm Children Who Do Not Cooperate for Preschool Developmental Testing. *JOURNAL OF PEDIATRICS* **1999**, *135*, 756–760, doi:10.1016/S0022-3476(99)70097-1.
127. Larsen, J.; Kochhar, P.; Wolke, D.; Draper, E.S.; Marlow, N.; Johnson, S. Comparing Behavioural Outcomes in Children Born Extremely Preterm between 2006 and 1995: The EPICure Studies. *Eur Child Adolesc Psychiatry* **2024**, *33*, 1517–1528, doi:10.1007/s00787-023-02258-w.
128. Lawrence, E.J.; Rubia, K.; Murray, R.M.; McGuire, P.K.; Walshe, M.; Allin, M.; Giampietro, V.; Rifkin, L.; Williams, S.C.R.; Nosarti, C. The Neural Basis of Response Inhibition and Attention Allocation as Mediated by Gestational Age. *HUMAN BRAIN MAPPING* **2009**, *30*, 1038–1050, doi:10.1002/hbm.20564.
129. LAWSON, K.; PARRINELLO, R.; RUFF, H. MATERNAL-BEHAVIOR AND INFANT ATTENTION. *INFANT BEHAVIOR & DEVELOPMENT* **1992**, *15*, 209–229, doi:10.1016/0163-6383(92)80024-O.
130. Lean, R.E.; Melzer, T.R.; Bora, S.; Watts, R.; Woodward, L.J. Attention and Regional Gray Matter Development in Very Preterm Children at Age 12 Years. *J Int Neuropsychol Soc* **2017**, *23*, 539–550, doi:10.1017/S1355617717000388.
131. Lean, R.E.; Lessov-Shlaggar, C.N.; Gerstein, E.D.; Smyser, T.A.; Paul, R.A.; Smyser, C.D.; Rogers, C.E. Maternal and Family Factors Differentiate Profiles of Psychiatric Impairments in Very Preterm Children at Age 5-Years. *J Child Psychol Psychiatry* **2020**, *61*, 157–166, doi:10.1111/jcpp.13116.
132. Lee, D.J.; Bryan, C.J.; Rudd, M.D. Longitudinal Suicide Ideation Trajectories in a Clinical Trial of Brief CBT for U.S. Military Personnel Recently Discharged from Psychiatric Hospitalization. *Psychiatry Res* **2020**, *293*, 113335, doi:10.1016/j.psychres.2020.113335.
133. Lee, S.W.; Guo, N.-W.; Huang, C.-C.; Huang, P.-C.; Chiang, C.-J.; Chien, Y.-H. Development of Cool and Hot Executive Function Deficit in Children Born Very Low Birth Weight with Normal Early Development: A Longitudinal Cohort from Aged 6 to 10. *EARLY HUMAN DEVELOPMENT* **2022**, *175*, doi:10.1016/j.earlhumdev.2022.105693.
134. Lejeune, F.; Lordier, L.; Pittet, M.P.; Schoenhals, L.; Grandjean, D.; Hüppi, P.S.; Filippa, M.; Borradori Tolsa, C. Effects of an Early Postnatal Music Intervention on Cognitive and Emotional Development in Preterm Children at 12 and 24 Months: Preliminary Findings. *Frontiers in Psychology* **2019**, *10*.
135. Lejeune, F.; Tolsa, C.B.; Graz, M.B.; Hueppi, P.S.; Barisnikov, K. Emotion, Attention, and Effortful Control in 24-Month-Old Very Preterm and Full-Term Children. *ANNEE PSYCHOLOGIQUE* **2015**, *115*, 241–264.
136. Leonhardt, M.; Forns, M.; Calderon, C.; Reinoso, M.; Gargallo, E. Visual Performance in Preterm Infants with Brain Injuries Compared with Low-Risk Preterm Infants. *EARLY HUMAN DEVELOPMENT* **2012**, *88*, 669–675, doi:10.1016/j.earlhumdev.2012.02.001.
137. Lindgren, M.; Stjernqvist, K.; Ors, M.; Rosén, I. Event-Related Potential Findings in Healthy Extremely Pre-Term (< Week 29) Children at Age 10. *Child Neuropsychol* **2000**, *6*, 77–86, doi:10.1076/chin.6.2.77.7054.
138. Litt, J.S.; Johnson, S.; Marlow, N.; Tiemeier, H. Impaired Pulmonary Function Mediates Inattention in Young Adults Born Extremely Preterm. *Acta Paediatr* **2023**, *112*, 254–260, doi:10.1111/apa.16586.

139. Litt, J.S.; Gerry Taylor, H.; Margevicius, S.; Schluchter, M.; Andreias, L.; Hack, M. Academic Achievement of Adolescents Born with Extremely Low Birth Weight. *Acta Paediatr* **2012**, *101*, 1240–1245, doi:10.1111/j.1651-2227.2012.02790.x.
140. Loe, I.M.; Lee, E.S.; Feldman, H.M. Attention and Internalizing Behaviors in Relation to White Matter in Children Born Preterm. *J Dev Behav Pediatr* **2013**, *34*, 156–164, doi:10.1097/DBP.0b013e3182842122.
141. Loe, I.M.; Lee, E.S.; Luna, F.; Feldman, H.M. Behavior Problems of 9-16 Year Old Preterm Children: Biological, Sociodemographic, and Intellectual Contributions. *EARLY HUMAN DEVELOPMENT* **2011**, *87*, 247–252, doi:10.1016/j.earlhumdev.2011.01.023.
142. Logan, J.W.; Dammann, O.; Allred, E.N.; Dammann, C.; Beam, K.; Joseph, R.M.; O'Shea, T.M.; Leviton, A.; Kuban, K.C.K. Early Postnatal Illness Severity Scores Predict Neurodevelopmental Impairments at 10 Years of Age in Children Born Extremely Preterm. *J Perinatol* **2017**, *37*, 606–614, doi:10.1038/jp.2016.242.
143. Loi, E.C.; Marchman, V.A.; Fernald, A.; Feldman, H.M. Using Eye Movements to Assess Language Comprehension in Toddlers Born Preterm and Full Term. *JOURNAL OF PEDIATRICS* **2017**, *180*, 124–129, doi:10.1016/j.jpeds.2016.10.004.
144. Lonnberg, P.; Pihko, E.; Lauronen, L.; Nurminen, J.; Andersson, S.; Metsaranta, M.; Lano, A.; Nevalainen, P. Secondary Somatosensory Cortex Evoked Responses and 6-Year Neurodevelopmental Outcome in Extremely Preterm Children. *CLINICAL NEUROPHYSIOLOGY* **2021**, *132*, 1572–1583, doi:10.1016/j.clinph.2021.04.005.
145. Ludyga, S.; Hanke, M.; Leuenberger, R.; Bruggisser, F.; Pühse, U.; Gerber, M.; Lemola, S.; Capone-Mori, A.; Keutler, C.; Brotzmann, M.; et al. Martial Arts and Cognitive Control in Children with Attention-Deficit Hyperactivity Disorder and Children Born Very Preterm: A Combined Analysis of Two Randomized Controlled Trials. *Med Sci Sports Exerc* **2023**, *55*, 777–786, doi:10.1249/MSS.0000000000003110.
146. Mahurin-Smith, J.; DeThorne, L.S.; Petrill, S.A. Children Born Prematurely May Demonstrate Catch-Up Growth in Pre-Adolescence. *LANGUAGE SPEECH AND HEARING SERVICES IN SCHOOLS* **2021**, *52*, 675–685, doi:10.1044/2020_LSHSS-20-00111.
147. Marlow, N.; Hennessy, E.M.; Bracewell, M.A.; Wolke, D.; EPICure Study Grp Motor and Executive Function at 6 Years of Age after Extremely Preterm Birth. *PEDIATRICS* **2007**, *120*, 793–804, doi:10.1542/peds.2007-0440.
148. McGrath, M.; Sullivan, M. Birth Weight, Neonatal Morbidities, and School Age Outcomes in Full-Term and Preterm Infants. *Issues Compr Pediatr Nurs* **2002**, *25*, 231–254, doi:10.1080/01460860290042611.
149. Mikkola, K.; Wetzel, N.; Leipala, J.; Serenius-Sirve, S.; Schroger, E.; Huotilainen, M.; Fellman, V. Behavioral and Evoked Potential Measures of Distraction in 5-Year-Old Children Born Preterm. *INTERNATIONAL JOURNAL OF PSYCHOPHYSIOLOGY* **2010**, *77*, 8–12, doi:10.1016/j.ijpsycho.2010.03.009.
150. Molloy, C.S.; Di Battista, A.M.; Anderson, V.A.; Burnett, A.; Lee, K.J.; Roberts, G.; Cheong, J.L.; Anderson, P.J.; Doyle, L.W. The Contribution of Visual Processing to Academic Achievement in Adolescents Born Extremely Preterm or Extremely Low Birth Weight. *CHILD NEUROPSYCHOLOGY* **2017**, *23*, 361–379, doi:10.1080/09297049.2015.1118024.
151. Morales, M.R.; Polizzi, C.; Sulliotti, G.; Mascolino, C.; Perricone, G. Early Precursors of Low Attention and Hyperactivity in Moderately and Very Preterm Children at Preschool Age. *Pediatr Rep* **2013**, *5*, e18, doi:10.4081/pr.2013.e18.
152. Morsing, E.; Lundgren, P.; Hard, A.-L.; Rakow, A.; Hellstrom-Westas, L.; Jacobson, L.; Johnson, M.; Nilsson, S.; Smith, L.E.H.; Savman, K.; et al. Neurodevelopmental Disorders and Somatic Diagnoses in a National Cohort of Children Born before 24 Weeks of Gestation. *ACTA PAEDIATRICA* **2022**, *111*, 1167–1175, doi:10.1111/apa.16316.
153. Morsing, E.; Asard, M.; Ley, D.; Stjernqvist, K.; Marsal, K. Cognitive Function After Intrauterine Growth Restriction and Very Preterm Birth. *PEDIATRICS* **2011**, *127*, E874–E882, doi:10.1542/peds.2010-1821.

154. Murner-Lavanchy, I.M.; Kidokoro, H.; Thompson, D.K.; Doyle, L.W.; Cheong, J.L.Y.; Hunt, R.W.; Inder, T.E.; Anderson, P.J. Thirteen-Year Outcomes in Very Preterm Children Associated with Diffuse Excessive High Signal Intensity on Neonatal Magnetic Resonance Imaging. *JOURNAL OF PEDIATRICS* **2019**, *206*, 66-+, doi:10.1016/j.jpeds.2018.10.016.
155. Murray, A.L.; Thompson, D.K.; Pascoe, L.; Leemans, A.; Inder, T.E.; Doyle, L.W.; Anderson, J.F.I.; Anderson, P.J. White Matter Abnormalities and Impaired Attention Abilities in Children Born Very Preterm. *NeuroImage* **2016**, *124*, 75–84, doi:10.1016/j.neuroimage.2015.08.044.
156. Nakagawa, A.; Sukigara, M.; Nomura, K.; Nagai, Y.; Miyachi, T. Attentional Differences in Audiovisual Face Perception between Full-and Preterm Very Low Birthweight Toddlers. *ACTA PAEDIATRICA* **2023**, *112*, 1715–1724, doi:10.1111/apa.16845.
157. Nelson, P.M.; Scheiber, F.; Laughlin, H.M.; Demir-Lira, O.E. Comparing Face-to-Face and Online Data Collection Methods in Preterm and Full-Term Children: An Exploratory Study. *FRONTIERS IN PSYCHOLOGY* **2021**, *12*, doi:10.3389/fpsyg.2021.733192.
158. Ni, T.-L.; Huang, C.-C.; Guo, N.-W. Executive Function Deficit in Preschool Children Born Very Low Birth Weight with Normal Early Development. *Early Human Development* **2011**, *87*, 137–141, doi:10.1016/j.earlhumdev.2010.11.013.
159. Nobre, F.D.; Gaspardo, C.M.; Linhares, M.B.M. Effortful Control and Attention as Predictors of Cognition in Children Born Preterm. *Clin Child Psychol Psychiatry* **2020**, *25*, 372–385, doi:10.1177/1359104519871652.
160. Ochiai, M.; Ichiyama, M.; Iwayama, M.; Sakai, Y.; Yoshida, K.; Hara, T. Longitudinal Study of Very Low Birth Weight Infants until 9years of Age; Attention Deficit Hyperactivity and Autistic Features Are Correlated with Their Cognitive Functions. *Early Hum Dev* **2015**, *91*, 783–786, doi:10.1016/j.earlhumdev.2015.09.005.
161. Odd, D.E.; Emond, A.; WhiTELaw, A. Long-Term Cognitive Outcomes of Infants Born Moderately and Late Preterm. *Developmental Medicine & Child Neurology* **2012**, *54*, 704–709, doi:10.1111/j.1469-8749.2012.04315.x.
162. O’Meagher, S.; Norris, K.; Kemp, N.; Anderson, P. Parent and Teacher Reporting of Executive Function and Behavioral Difficulties in Preterm and Term Children at Kindergarten. *Applied Neuropsychology: Child* **2020**, *9*, 153–164, doi:10.1080/21622965.2018.1550404.
163. Oosterom, L.; Bogicevic, L.; Verhoeven, M.; van Baar, A.L. Parenting Behavior at 18 Months Predicts Internalizing and Externalizing Problems at 6 Years in Moderately Preterm and Full Term Children. *INTERNATIONAL JOURNAL OF ENVIRONMENTAL RESEARCH AND PUBLIC HEALTH* **2020**, *17*, doi:10.3390/ijerph17228679.
164. O’Shea, T.M.; Joseph, R.M.; Kuban, K.C.K.; Allred, E.N.; Ware, J.; Coster, T.; Fichorova, R.N.; Dammann, O.; Leviton, A.; ELGAN Study Investigators Elevated Blood Levels of Inflammation-Related Proteins Are Associated with an Attention Problem at Age 24 Mo in Extremely Preterm Infants. *PEDIATRIC RESEARCH* **2014**, *75*, 781–787, doi:10.1038/pr.2014.41.
165. Ostgard, H.F.; Solsnes, A.E.; Bjuland, K.J.; Rimol, L.M.; Martinussen, M.; Brubakk, A.-M.; Haberg, A.K.; Skranes, J.; Lohaugen, G.C.C. Executive Function Relates to Surface Area of Frontal and Temporal Cortex in Very-Low-Birth-Weight Late Teenagers. *EARLY HUMAN DEVELOPMENT* **2016**, *95*, 47–53, doi:10.1016/j.earlhumdev.2016.01.023.
166. Padilla, N.; Escrichs, A.; del Agua, E.; Kringelbach, M.; Donaire, A.; Deco, G.; Åden, U. Disrupted Resting-Sate Brain Network Dynamics in Children Born Extremely Preterm. *Cereb Cortex* **2023**, *33*, 8101–8109, doi:10.1093/cercor/bhad101.
167. Palumbi, R.; Peschechera, A.; Margari, M.; Craig, F.; Cristella, A.; Petruzzelli, M.G.; Margari, L. Neurodevelopmental and Emotional-Behavioral Outcomes in Late-Preterm Infants: An Observational Descriptive Case Study. *BMC PEDIATRICS* **2018**, *18*, doi:10.1186/s12887-018-1293-6.
168. Pascoe, L.; Roberts, G.; Doyle, L.W.; Lee, K.J.; Thompson, D.K.; Seal, M.L.; Josev, E.K.; Nosarti, C.; Gathercole, S.; Anderson, P.J. Preventing Academic Difficulties in Preterm Children: A Randomised Controlled Trial of an Adaptive Working Memory Training Intervention - IMPRINT Study. *BMC PEDIATRICS* **2013**, *13*, doi:10.1186/1471-2431-13-144.

169. Fernández, C.P.; Cánovas, R.; Moreno-Montoya, M.; Sánchez, F.S.; Cubos, P.F. Go/NoGo Training Improves Executive Functions in an 8-Year-Old Child Born Preterm. *Revista de Psicología Clínica con Niños y Adolescentes* **2017**.
170. Perricone, G.; Morales, M.R.; Anzalone, G. Neurodevelopmental Outcomes of Moderately Preterm Birth: Precursors of Attention Deficit Hyperactivity Disorder at Preschool Age. *Springerplus* **2013**, *2*, 221, doi:10.1186/2193-1801-2-221.
171. Perricone, G.; Morales, M.R. The Temperament of Preterm Infant in Preschool Age. *Ital J Pediatr* **2011**, *37*, 4, doi:10.1186/1824-7288-37-4.
172. Petrie Thomas, J.H.; Whitfield, M.F.; Oberlander, T.F.; Synnes, A.R.; Grunau, R.E. Focused Attention, Heart Rate Deceleration, and Cognitive Development in Preterm and Full-Term Infants. *DEVELOPMENTAL PSYCHOBIOLOGY* **2012**, *54*, 383–400, doi:10.1002/dev.20597.
173. Pierrat, V.; Marchand-Martin, L.; Marret, S.; Arnaud, C.; Benhammou, V.; Cambonie, G.; Debillon, T.; Dufourg, M.-N.; Gire, C.; Goffinet, F.; et al. Neurodevelopmental Outcomes at Age 5 among Children Born Preterm: EPIPAGE-2 Cohort Study. *BMJ-BRITISH MEDICAL JOURNAL* **2021**, *373*, doi:10.1136/bmj.n741.
174. Pizzo, R.; Urben, S.; Linden, M.V.D.; Borradori-Tolsa, C.; Freschi, M.; Forcada-Guex, M.; Hüppi, P.; Barisnikov, K. Attentional Networks Efficiency in Preterm Children. *Journal of the International Neuropsychological Society* **2010**, *16*, 130–137, doi:10.1017/S1355617709991032.
175. Poehlmann, J.; Schwichtenberg, A.J.M.; Shah, P.E.; Shlafer, R.J.; Hahn, E.; Maleck, S. The Development of Effortful Control in Children Born Preterm. *JOURNAL OF CLINICAL CHILD AND ADOLESCENT PSYCHOLOGY* **2010**, *39*, 522–536, doi:10.1080/15374416.2010.486319.
176. Purdy, I.B.; Smith, L.; Wiley, D.; Badr, L. A Psychoneuroimmunologic Examination of Cumulative Perinatal Steroid Exposures and Preterm Infant Behavioral Follow-Up. *BIOLOGICAL RESEARCH FOR NURSING* **2013**, *15*, 86–95, doi:10.1177/1099800411420134.
177. Rand, K.M.; Austin, N.C.; Inder, T.E.; Bora, S.; Woodward, L.J. Neonatal Infection and Later Neurodevelopmental Risk in the Very Preterm Infant. *J Pediatr* **2016**, *170*, 97–104, doi:10.1016/j.jpeds.2015.11.017.
178. Reid, L.D.; Strobino, D.M. A Population-Based Study of School Readiness Determinants in a Large Urban Public School District. *MATERNAL AND CHILD HEALTH JOURNAL* **2019**, *23*, 325–334, doi:10.1007/s10995-018-2666-z.
179. Reijneveld, S.A.; Hornman, J.; Boelema, S.R.; de Winter, A.F. Stability of Executive Functioning of Moderately-Late Preterm and Full-Term Born Children at Ages 11 and 19: The TRAILS Cohort Study. *Int J Environ Res Public Health* **2021**, *18*, 4161, doi:10.3390/ijerph18084161.
180. Reijneveld, S.A.; de Kleine, M.J.K.; van Baar, A.L.; Kollee, L.A.A.; Verhaak, C.M.; Verhulst, F.C.; Verloove-Vanhorick, S.P. Behavioural and Emotional Problems in Very Preterm and Very Low Birthweight Infants at Age 5 Years. *ARCHIVES OF DISEASE IN CHILDHOOD-FETAL AND NEONATAL EDITION* **2006**, *91*, F423–F428, doi:10.1136/adc.2006.093674.
181. Retzler, J.; Retzler, C.; Groom, M.; Johnson, S.; Cragg, L. Using Drift Diffusion Modeling to Understand Inattentive Behavior in Preterm and Term-Born Children. *NEUROPSYCHOLOGY* **2020**, *34*, 77–87, doi:10.1037/neu0000590.
182. Retzler, J.; Johnson, S.; Groom, M.; Hollis, C.; Budge, H.; Cragg, L. Cognitive Predictors of Parent-Rated Inattention in Very Preterm Children: The Role of Working Memory and Processing Speed. *Child Neuropsychol* **2019**, *25*, 617–635, doi:10.1080/09297049.2018.1510908.
183. Reuner, G.; Weinschenk, A.; Pauen, S.; Pietz, J. Cognitive Development in 7- to 24-Month-Old Extremely/Very-to-Moderately/Late Preterm and Full-Term Born Infants: The Mediating Role of Focused Attention. *Child Neuropsychol* **2015**, *21*, 314–330, doi:10.1080/09297049.2014.899571.
184. Reveillon, M.; Tolsa, C.B.; Monnier, M.; Hueppi, P.S.; Barisnikov, K. Response Inhibition Difficulties in Preterm Children Aged 9-12 Years: Relations with Emotion and Behavior. *CHILD NEUROPSYCHOLOGY* **2016**, *22*, 420–442, doi:10.1080/09297049.2014.994486.
185. Reveillon, M.; Urben, S.; Barisnikov, K.; Tolsa, C.B.; Hueppi, P.S.; Lazeyras, F. Functional Neuroimaging Study of Performances on a Go/No-Go Task in 6-to 7-Year-Old Preterm Children:

Impact of Intrauterine Growth Restriction. *NEUROIMAGE-CLINICAL* **2013**, *3*, 429–437, doi:10.1016/j.nicl.2013.10.007.

186. Ribeiro, L.A.; Zachrisson, H.D.; Schjolberg, S.; Aase, H.; Rohrer-Baumgartner, N.; Magnus, P. Attention Problems and Language Development in Preterm Low-Birth-Weight Children: Cross-Lagged Relations from 18 to 36 Months. *BMC Pediatrics* **2011**, *11*, 59, doi:10.1186/1471-2431-11-59.
187. Ritter, B.C.; Perrig, W.; Steinlin, M.; Everts, R. Cognitive and Behavioral Aspects of Executive Functions in Children Born Very Preterm. *CHILD NEUROPSYCHOLOGY* **2014**, *20*, 129–144, doi:10.1080/09297049.2013.773968.
188. Rose, S.A.; Feldman, J.F.; Jankowski, J.J. Information Processing in Toddlers: Continuity from Infancy and Persistence of Preterm Deficits. *Intelligence* **2009**, *37*, 311–320, doi:10.1016/j.intell.2009.02.002.
189. Ross, G.S.; Perlman, J.M. Relationship of Withdrawn, Anxious/Depressed and Attention Problems to Cognitive Performance in Preterm Children at 18 Months and 36 Months of Age. *Infant Behav Dev* **2022**, *66*, 101660, doi:10.1016/j.infbeh.2021.101660.
190. Ross-Sheehy, S.; Perone, S.; Macek, K.L.; Eschman, B. Visual Orienting and Attention Deficits in 5- and 10-Month-Old Preterm Infants. *Infant Behav Dev* **2017**, *46*, 80–90, doi:10.1016/j.infbeh.2016.12.004.
191. Roze, E.; Reijneveld, S.A.; Stewart, R.E.; Bos, A.F. Multi-Domain Cognitive Impairments at School Age in Very Preterm-Born Children Compared to Term-Born Peers. *BMC Pediatrics* **2021**, *21*, 169, doi:10.1186/s12887-021-02641-z.
192. RUFF, H.; LAWSON, K.; PARRINELLO, R.; WEISSBERG, R. LONG-TERM STABILITY OF INDIVIDUAL-DIFFERENCES IN SUSTAINED ATTENTION IN THE EARLY YEARS. *CHILD DEVELOPMENT* **1990**, *61*, 60–75.
193. Rushe, T.M.; Rifkin, L.; Stewart, A.L.; Townsend, J.P.; Roth, S.C.; Wyatt, J.S.; Murray, R.M. Neuropsychological Outcome at Adolescence of Very Preterm Birth and Its Relation to Brain Structure. *Dev Med Child Neurol* **2001**, *43*, 226–233, doi:10.1017/s0012162201000433.
194. Ruys, C.A.; Bröring, T.; van Schie, P.E.M.; van de Lagemaat, M.; Rotteveel, J.; Finken, M.J.J.; Oostrom, K.J.; Lafeber, H.N. Neurodevelopment of Children Born Very Preterm and/or with a Very Low Birth Weight: 8-Year Follow-up of a Nutritional RCT. *Clin Nutr ESPEN* **2019**, *30*, 190–198, doi:10.1016/j.clnesp.2018.12.083.
195. Ryu, H.; Han, G.; Choi, J.; Park, H.-K.; Kim, M.J.; Ahn, D.-H.; Lee, H.J. Object Permanence and the Development of Attention Capacity in Preterm and Term Infants: An Eye-Tracking Study. *Italian Journal of Pediatrics* **2017**, *43*, 90, doi:10.1186/s13052-017-0408-2.
196. Sajaniemi, N.; Salokorpi, T.; von Wendt, L. Temperament Profiles and Their Role in Neurodevelopmental Assessed Preterm Children at Two Years of Age. *Eur Child Adolesc Psychiatry* **1998**, *7*, 145–152, doi:10.1007/s007870050060.
197. Salavati, S.; Bos, A.F.; Doyle, L.W.; Anderson, P.J.; Spittle, A.J. Very Preterm Early Motor Repertoire and Neurodevelopmental Outcomes at 8 Years. *PEDIATRICS* **2021**, *148*, doi:10.1542/peds.2020-049572.
198. Samara, M.; Marlow, N.; Wolke, D.; EPIcure Study Grp Pervasive Behavior Problems at 6 Years of Age in a Total-Population Sample of Children Born at ≤25 Weeks of Gestation. *PEDIATRICS* **2008**, *122*, 562–573, doi:10.1542/peds.2007-3231.
199. Samuelsson, M.; Holsti, A.; Adamsson, M.; Serenius, F.; Hagglof, B.; Farooqi, A. Behavioral Patterns in Adolescents Born at 23 to 25 Weeks of Gestation. *PEDIATRICS* **2017**, *140*, doi:10.1542/peds.2017-0199.
200. Sansavini, A.; Zavagli, V.; Guarini, A.; Savini, S.; Alessandroni, R.; Faldella, G. Dyadic Co-Regulation, Affective Intensity and Infant's Development at 12 Months: A Comparison among Extremely Preterm and Full-Term Dyads. *INFANT BEHAVIOR & DEVELOPMENT* **2015**, *40*, 29–40, doi:10.1016/j.infbeh.2015.03.005.
201. Schieve, L.A.; Tian, L.H.; Rankin, K.; Kogan, M.D.; Yeargin-Allsopp, M.; Visser, S.; Rosenberg, D. Population Impact of Preterm Birth and Low Birth Weight on Developmental Disabilities in US Children. *Ann Epidemiol* **2016**, *26*, 267–274, doi:10.1016/j.annepidem.2016.02.012.

202. Schmidt, C.; Lawson, K. Caregiver Attention-Focusing and Children's Attention-Sharing Behaviours as Predictors of Later Verbal IQ in Very Low Birthweight Children. *JOURNAL OF CHILD LANGUAGE* **2002**, *29*, 3–22, doi:10.1017/S0305000901004913.
203. Schothorst, P.; VanEngeland, H. Long-Term Behavioral Sequelae of Prematurity. *JOURNAL OF THE AMERICAN ACADEMY OF CHILD AND ADOLESCENT PSYCHIATRY* **1996**, *35*, 175–183, doi:10.1097/00004583-199602000-00011.
204. Schwichtenberg, A.J.; Christ, S.; Abel, E.; Poehlmann-Tynan, J.A. Circadian Sleep Patterns in Toddlers Born Preterm: Longitudinal Associations with Developmental and Health Concerns. *JOURNAL OF DEVELOPMENTAL AND BEHAVIORAL PEDIATRICS* **2016**, *37*, 358–369, doi:10.1097/DBP.0000000000000287.
205. Scott, A.; Winchester, S.B.; Sullivan, M.C. Trajectories of Problem Behaviors from 4 to 23 Years in Former Preterm Infants. *INTERNATIONAL JOURNAL OF BEHAVIORAL DEVELOPMENT* **2018**, *42*, 237–247, doi:10.1177/0165025417692899.
206. Scratch, S.E.; Anderson, P.J.; Doyle, L.W.; Thompson, D.K.; Ahmadzai, Z.M.; Greaves, R.F.; Inder, T.E.; Hunt, R.W. High Postnatal Growth Hormone Levels Are Related to Cognitive Deficits in a Group of Children Born Very Preterm. *J Clin Endocrinol Metab* **2015**, *100*, 2709–2717, doi:10.1210/jc.2014-4342.
207. Sekigawa-Hosozawa, M.; Tanaka, K.; Shimizu, T.; Nakano, T.; Kitazawa, S. A Group of Very Preterm Children Characterized by Atypical Gaze Patterns. *BRAIN & DEVELOPMENT* **2017**, *39*, 218–224, doi:10.1016/j.braindev.2016.10.001.
208. Serenius, F.; Kaul, Y.F.; Kallen, K.; Hafstrom, M.; Aden, U.; Stjernqvist, K.; Farooqi, A.; EXPRESS Study Grp Neurobehavioral Symptoms in Children Born Extremely Preterm: A Swedish National Study. *ACTA PAEDIATRICA* **2023**, *112*, 2387–2399, doi:10.1111/apa.16942.
209. Shaw, J.C.; Berry, M.J.; Dyson, R.M.; Crombie, G.K.; Hirst, J.J.; Palliser, H.K. Reduced Neurosteroid Exposure Following Preterm Birth and Its' Contribution to Neurological Impairment: A Novel Avenue for Preventative Therapies. *Front Physiol* **2019**, *10*, 599, doi:10.3389/fphys.2019.00599.
210. Shi, J.; Wang, Y.; Ceschin, R.; An, X.; Lao, Y.; Vanderbilt, D.; Nelson, M.D.; Thompson, P.M.; Panigrahy, A.; Lepore, N. A Multivariate Surface-Based Analysis of the Putamen in Premature Newborns: Regional Differences within the Ventral Striatum. *PLOS ONE* **2013**, *8*, doi:10.1371/journal.pone.0066736.
211. Shih, H.-N.; Tsai, W.-H.; Chang, S.-H.; Lin, C.-Y.; Hong, R.-B.; Hwang, Y.-S. Chinese Handwriting Performance in Preterm Children in Grade 2. *PLOS ONE* **2018**, *13*, doi:10.1371/journal.pone.0199355.
212. Shinya, Y.; Kawai, M.; Niwa, F.; Kanakogi, Y.; Imafuku, M.; Myowa, M. Cognitive Flexibility in 12-Month-Old Preterm and Term Infants Is Associated with Neurobehavioural Development in 18-Month-Olds. *SCIENTIFIC REPORTS* **2022**, *12*, doi:10.1038/s41598-021-04194-8.
213. SIGMAN, M.; COHEN, S.; BECKWITH, L.; PARMELEE, A. INFANT ATTENTION IN RELATION TO INTELLECTUAL ABILITIES IN CHILDHOOD. *DEVELOPMENTAL PSYCHOLOGY* **1986**, *22*, 788–792, doi:10.1037/0012-1649.22.6.788.
214. Simon, L.; Nusinovici, S.; Flamant, C.; Cariou, B.; Rouger, V.; Gascoin, G.; Darmaun, D.; Roze, J.-C.; Hanf, M. Post-Term Growth and Cognitive Development at 5 Years of Age in Preterm Children: Evidence from a Prospective Population-Based Cohort. *PLOS ONE* **2017**, *12*, doi:10.1371/journal.pone.0174645.
215. Smith, K.; Landry, S.; Swank, P.; Baldwin, C.; Denson, S.; Wildin, S. The Relation of Medical Risk and Maternal Stimulation with Preterm Infants' Development of Cognitive, Language and Daily Living Skills. *JOURNAL OF CHILD PSYCHOLOGY AND PSYCHIATRY* **1996**, *37*, 855–864, doi:10.1111/j.1469-7610.1996.tb01481.x.
216. Smyrni, N.; Koutsaki, M.; Petra, M.; Nikaina, E.; Gontika, M.; Strataki, H.; Davora, F.; Bouza, H.; Damianos, G.; Skouteli, H.; et al. Moderately and Late Preterm Infants: Short- and Long-Term Outcomes From a Registry-Based Cohort. *FRONTIERS IN NEUROLOGY* **2021**, *12*, doi:10.3389/fneur.2021.628066.

217. Snijders, V.E.; Bogicevic, L.; Verhoeven, M.; van Baar, A.L. Toddlers' Language Development: The Gradual Effect of Gestational Age, Attention Capacities, and Maternal Sensitivity. *INTERNATIONAL JOURNAL OF ENVIRONMENTAL RESEARCH AND PUBLIC HEALTH* **2020**, *17*, doi:10.3390/ijerph17217926.
218. Squarza, C.; Gardon, L.; Gianni, M.L.; Frigerio, A.; Gangi, S.; Porro, M.; Mosca, F.; Picciolini, O. Neurodevelopmental Outcome and Adaptive Behavior in Preterm Multiples and Singletons at 1 and 2 Years of Corrected Age. *FRONTIERS IN PSYCHOLOGY* **2020**, *11*, doi:10.3389/fpsyg.2020.01653.
219. Stedall, P.M.; Spencer-Smith, M.M.; Mainzer, R.M.; Treyvaud, K.; Burnett, A.C.; Doyle, L.W.; Spittle, A.J.; Anderson, P.J. Thirteen-Year Outcomes of a Randomized Clinical Trial of Early Preventive Care for Very Preterm Infants and Their Parents. *The Journal of Pediatrics* **2022**, *246*, 80-88.e4, doi:10.1016/j.jpeds.2022.03.013.
220. Stedall, P.M.; Spencer-Smith, M.M.; Lah, S.; Doyle, L.W.; Spittle, A.J.; Burnett, A.C.; Anderson, P.J. Episodic and Prospective Memory Difficulties in 13-Year-Old Children Born Very Preterm. *JOURNAL OF THE INTERNATIONAL NEUROPSYCHOLOGICAL SOCIETY* **2023**, *29*, 257–265, doi:10.1017/S1355617722000170.
221. Stjernqvist, K.; Svenningsen, N.W. Ten-Year Follow-up of Children Born before 29 Gestational Weeks: Health, Cognitive Development, Behaviour and School Achievement. *Acta Paediatr* **1999**, *88*, 557–562, doi:10.1080/08035259950169594.
222. Strang-Karlsson, S.; Andersson, S.; Paile-Hyvarinen, M.; Darby, D.; Hovi, P.; Raikkonen, K.; Pesonen, A.-K.; Heinonen, K.; Jarvenpaa, A.-L.; Eriksson, J.G.; et al. Slower Reaction Times and Impaired Learning in Young Adults With Birth Weight <1500 g. *PEDIATRICS* **2010**, *125*, E74–E82, doi:10.1542/peds.2009-1297.
223. Stroustrup, A.; Bragg, J.B.; Andra, S.S.; Curtin, P.C.; Spear, E.A.; Sison, D.B.; Just, A.C.; Arora, M.; Gennings, C. Neonatal Intensive Care Unit Phthalate Exposure and Preterm Infant Neurobehavioral Performance. *PLOS ONE* **2018**, *13*, doi:10.1371/journal.pone.0193835.
224. Suikkanen, J.; Miettola, S.; Heinonen, K.; Vaarasmaki, M.; Tikanmaki, M.; Sipola, M.; Matinolli, H.-M.; Jarvelin, M.-R.; Raikkonen, K.; Hovi, P.; et al. Reaction Times, Learning, and Executive Functioning in Adults Born Preterm. *PEDIATRIC RESEARCH* **2021**, *89*, 198–204, doi:10.1038/s41390-020-0851-4.
225. Sun, J.; Buys, N. A Comparison of Sustained Attention in Very Preterm and Term Infants. *International Journal of Child and Adolescent Health* **2012**, *5*, 291–300.
226. Sun, J.; Mohay, H.; O'Callaghan, M. A Comparison of Executive Function in Very Preterm and Term Infants at 8 Months Corrected Age. *EARLY HUMAN DEVELOPMENT* **2009**, *85*, 225–230, doi:10.1016/j.earlhumdev.2008.10.005.
227. Takeuchi, A.; Ogino, T.; Koeda, T.; Oka, M.; Yorifuji, T.; Takayanagi, T.; Sato, K.; Sugino, N.; Bonno, M.; Nakamura, M.; et al. Intelligence Test at Preschool-Age Predicts Reading Difficulty among School-Aged Very Low Birth Weight Infants in Japan. *BRAIN & DEVELOPMENT* **2018**, *40*, 735–742, doi:10.1016/j.braindev.2018.05.002.
228. Takeuchi, A.; Yorifuji, T.; Hattori, M.; Tamai, K.; Nakamura, K.; Nakamura, M.; Kageyama, M.; Kubo, T.; Ogino, T.; Kobayashi, K.; et al. Catch-up Growth and Behavioral Development among Preterm, Small-for-Gestational-Age Children: A Nationwide Japanese Population-Based Study. *BRAIN & DEVELOPMENT* **2019**, *41*, 397–405, doi:10.1016/j.braindev.2018.12.004.
229. Talge, N.M.; Holzman, C.; Van Egeren, L.A.; Symonds, L.L.; Scheid, J.M.; Senagore, P.K.; Sikorskii, A. Late-Preterm Birth by Delivery Circumstance and Its Association with Parent-Reported Attention Problems in Childhood. *J Dev Behav Pediatr* **2012**, *33*, 405–415, doi:10.1097/DBP.0b013e3182564704.
230. Talge, N.M.; Holzman, C.; Wang, J.; Lucia, V.; Gardiner, J.; Breslau, N. Late-Preterm Birth and Its Association With Cognitive and Socioemotional Outcomes at 6 Years of Age. *OBSTETRICAL & GYNECOLOGICAL SURVEY* **2011**, *66*, 193–195, doi:10.1097/OGX.0b013e318225c476.
231. Tanaka, K.; Hosozawa, M.; Kudo, N.; Yoshikawa, N.; Hisata, K.; Shoji, H.; Shinohara, K.; Shimizu, T. The Pilot Study: Sphingomyelin-Fortified Milk Has a Positive Association with the

Neurobehavioural Development of Very Low Birth Weight Infants during Infancy, Randomized Control Trial. *BRAIN & DEVELOPMENT* **2013**, *35*, 45–52, doi:10.1016/j.braindev.2012.03.004.
232. Tanis, J.C.; van der Ree, M.H.; Roze, E.; Huis in ‘t Veld, A.E.; van den Berg, P.P.; Van Braeckel, K.N.J.A.; Bos, A.F. Functional Outcome of Very Preterm–Born and Small-for-Gestational-Age Children at School Age. *Pediatr Res* **2012**, *72*, 641–648, doi:10.1038/pr.2012.130.
233. Thiriez, G.; Mougey, C.; Vermeylen, D.; Wermenbol, V.; Lanquart, J.-P.; Lin, J.S.; Franco, P. Altered Autonomic Control in Preterm Newborns with Impaired Neurological Outcomes. *CLINICAL AUTONOMIC RESEARCH* **2015**, *25*, 233–242, doi:10.1007/s10286-015-0298-6.
234. Thompson, D.K.; Yang, J.Y.M.; Chen, J.; Kelly, C.E.; Adamson, C.L.; Alexander, B.; Gilchrist, C.; Matthews, L.G.; Lee, K.J.; Hunt, R.W.; et al. Brain White Matter Development Over the First 13 Years in Very Preterm and Typically Developing Children Based on the T1-w/T2-w Ratio. *NEUROLOGY* **2022**, *98*, E924–E937, doi:10.1212/WNL.0000000000013250.
235. Tideman, E. Longitudinal Follow-up of Children Born Preterm: Cognitive Development at Age 19. *EARLY HUMAN DEVELOPMENT* **2000**, *58*, 81–90, doi:10.1016/S0378-3782(00)00055-4.
236. Toijonen, A.; Heinonen, S.; Gissler, M.; Seikku, L.; Macharey, G. Impact of Fetal Presentation on Neurodevelopmental Outcome in a Trial of Preterm Vaginal Delivery: A Nationwide, Population-Based Record Linkage Study. *ARCHIVES OF GYNECOLOGY AND OBSTETRICS* **2022**, *306*, 29–35, doi:10.1007/s00404-021-06146-z.
237. Tommiska, V.; Lano, A.; Kleemola, P.; Klenberg, L.; Lehtonen, L.; Löppönen, T.; Olsen, P.; Tammela, O.; Fellman, V.; Finnish ELBW Cohort Study Group (FinELBW) Analysis of Neurodevelopmental Outcomes of Preadolescents Born with Extremely Low Weight Revealed Impairments in Multiple Developmental Domains despite Absence of Cognitive Impairment. *Health Sci Rep* **2020**, *3*, e180, doi:10.1002/hsr2.180.
238. Torrioli, M.; Frisone, M.; Bonvini, L.; Luciano, R.; Pasca, M.; Lepori, R.; Tortorolo, G.; Guzzetta, F. Perceptual-Motor, Visual and Cognitive Ability in Very Low Birthweight Preschool Children without Neonatal Ultrasound Abnormalities. *BRAIN & DEVELOPMENT* **2000**, *22*, 163–168, doi:10.1016/S0387-7604(00)00098-X.
239. Trickett, J.; Bernardi, M.; Fahy, A.; Lancaster, R.; Larsen, J.; Ni, Y.; Suonpera, E.; Wolke, D.; Marlow, N.; Johnson, S. Neuropsychological Abilities Underpinning Academic Attainment in Children Born Extremely Preterm. *CHILD NEUROPSYCHOLOGY* **2022**, *28*, 746–767, doi:10.1080/09297049.2021.2014433.
240. Tseng, C.-E.J.; Pascoe, L.; Roberts, G.; Doyle, L.W.; Lee, K.J.; Thompson, D.K.; Seal, M.; Josev, E.K.; Chen, J.; Nosarti, C.; et al. Working Memory Training Is Associated with Changes in Resting State Functional Connectivity in Children Who Were Born Extremely Preterm: A Randomized Controlled Trial. *JOURNAL OF COGNITIVE ENHANCEMENT* **2019**, *3*, 376–387, doi:10.1007/s41465-019-00150-7.
241. Twilhaar, E.S.; Belopolsky, A.V.; de Kieviet, J.F.; van Elburg, R.M.; Oosterlaan, J. Voluntary and Involuntary Control of Attention in Adolescents Born Very Preterm: A Study of Eye Movements. *CHILD DEVELOPMENT* **2020**, *91*, 1272–1283, doi:10.1111/cdev.13310.
242. Twilhaar, E.S.; De Kieviet, J.F.; Van Elburg, R.M.; Oosterlaan, J. Neurocognitive Processes Underlying Academic Difficulties in Very Preterm Born Adolescents. *CHILD NEUROPSYCHOLOGY* **2020**, *26*, 274–287, doi:10.1080/09297049.2019.1639652.
243. Twilhaar, E.S.; de Kieviet, J.F.; Oosterlaan, J.; van Elburg, R.M. A Randomised Trial of Enteral Glutamine Supplementation for Very Preterm Children Showed No Beneficial or Adverse Long-Term Neurodevelopmental Outcomes. *Acta Paediatr* **2018**, *107*, 593–599, doi:10.1111/apa.14167.
244. Urben, S.; De Jonge, L.V.H.; Barisnikov, K.; Pizzo, R.; Monnier, M.; Lazeyras, F.; Tolsa, C.B.; Huppi, P.S. Gestational Age and Gender Influence on Executive Control and Its Related Neural Structures in Preterm-Born Children at 6 Years of Age. *CHILD NEUROPSYCHOLOGY* **2017**, *23*, 188–207, doi:10.1080/09297049.2015.1099619.
245. van Baar, A.L.; de Jong, M.; Maat, M.; Hooge, I.T.C.; Bogicevic, L.; Verhoeven, M. Reliability and Validity of the Utrecht Tasks for Attention in Toddlers Using Eye Tracking (UTATE). *FRONTIERS IN PSYCHOLOGY* **2020**, *11*, doi:10.3389/fpsyg.2020.01179.

246. van der Burg, J.W.; Jensen, E.T.; van de Bor, M.; Joseph, R.M.; O'Shea, T.M.; Kuban, K.; Allred, E.N.; Scott, M.; Hunter, S.; Hooper, S.R.; et al. Maternal Obesity and Attention-Related Symptoms in the Preterm Offspring. *Early Hum Dev* **2017**, *115*, 9–15, doi:10.1016/j.earlhumdev.2017.08.002.
247. van der Ree, M.; Tanis, J.C.; Van Braeckel, K.N.J.A.; Bos, A.F.; Roze, E. Functional Impairments at School Age of Preterm Born Children with Late-Onset Sepsis. *Early Hum Dev* **2011**, *87*, 821–826, doi:10.1016/j.earlhumdev.2011.06.008.
248. van Gils, M.M.; Dudink, J.; Reiss, I.K.M.; Swarte, R.M.C.; van der Steen, J.; Pel, J.J.M.; Kooiker, M.J.G. Brain Damage and Visuospatial Impairments: Exploring Early Structure-Function Associations in Children Born Very Preterm. *PEDIATRIC NEUROLOGY* **2020**, *109*, 63–71, doi:10.1016/j.pediatrneurol.2019.12.010.
249. van Houdt, C.A.; Aarnoudse-Moens, C.S.H.; van Wassenaer-Leemhuis, A.G.; Laarman, A.R.C.; Koopman-Esseboom, C.; van Kaam, A.H.; Oosterlaan, J. Effects of Executive Function Training on Attentional, Behavioral and Emotional Functioning and Self-Perceived Competence in Very Preterm Children: A Randomized Controlled Trial. *Front Psychol* **2019**, *10*, 2100, doi:10.3389/fpsyg.2019.02100.
250. van Houdt, C.A.; van Wassenaer-Leemhuis, A.G.; Oosterlaan, J.; Königs, M.; Koopman-Esseboom, C.; Laarman, A.R.C.; van Kaam, A.H.; Aarnoudse-Moens, C.S.H. Executive Function Training in Very Preterm Children: A Randomized Controlled Trial. *Eur Child Adolesc Psychiatry* **2021**, *30*, 785–797, doi:10.1007/s00787-020-01561-0.
251. van Houdt, C.A.; van Wassenaer-Leemhuis, A.G.; Oosterlaan, J.; van Kaam, A.H.; Aarnoudse-Moens, C.S.H. Developmental Outcomes of Very Preterm Children with High Parental Education Level. *EARLY HUMAN DEVELOPMENT* **2019**, *133*, 11–17, doi:10.1016/j.earlhumdev.2019.04.010.
252. van Houdt, C.A.; Oosterlaan, J.; Aarnoudse-Moens, C.S.H.; van Kaam, A.H.; van Wassenaer-Leemhuis, A.G. Subtypes of Behavioral Functioning in 8-12 Year Old Very Preterm Children. *EARLY HUMAN DEVELOPMENT* **2020**, *142*, doi:10.1016/j.earlhumdev.2020.104968.
253. Van Hus, J.W.; Potharst, E.S.; Jeukens-Visser, M.; Kok, J.H.; Van Wassenaer-Leemhuis, A.G. Motor Impairment in Very Preterm-Born Children: Links with Other Developmental Deficits at 5 Years of Age. *DEVELOPMENTAL MEDICINE AND CHILD NEUROLOGY* **2014**, *56*, 587–594, doi:10.1111/dmcn.12295.
254. Vanderbilt, D.; Gleason, M.M. Mental Health Concerns of the Premature Infant Through the Lifespan. *PEDIATRIC CLINICS OF NORTH AMERICA* **2011**, *58*, 815-+, doi:10.1016/j.pcl.2011.06.012.
255. Vederhus, B.J.; Markestad, T.; Eide, G.E.; Graue, M.; Halvorsen, T. Health Related Quality of Life after Extremely Preterm Birth: A Matched Controlled Cohort Study. *HEALTH AND QUALITY OF LIFE OUTCOMES* **2010**, *8*, doi:10.1186/1477-7525-8-53.
256. Verkerk, G.; Jeukens-Visser, M.; Houtzager, B.; Wassenaer-Leemhuis, A. van; Koldewijn, K.; Nollet, F.; Kok, J. Attention in 3-Year-Old Children with VLBW and Relationships with Early School Outcomes. *Phys Occup Ther Pediatr* **2016**, *36*, 59–72, doi:10.3109/01942638.2015.1012319.
257. Walczak-Kozłowska, T.; Chrzan-Dętkoś, M.; Harciarek, M. Heterogeneity of the Attentional System's Efficiency among Very Prematurely Born Pre-Schoolers. *Child Neuropsychology* **2022**, *28*, 120–142, doi:10.1080/09297049.2021.1961702.
258. Walczak-Kozłowska, T.; Mańkowska, A.; Chrzan-Dętkoś, M.; Harciarek, M. Attentional System of Very Prematurely Born Preschoolers. *Dev Psychol* **2020**, *56*, 251–260, doi:10.1037/dev0000865.
259. Wehrle, F.M.; Michels, L.; Guggenberger, R.; Huber, R.; Latal, B.; O'Gorman, R.L.; Hagmann, C.F. Altered Resting-State Functional Connectivity in Children and Adolescents Born Very Preterm Short Title. *NEUROIMAGE-CLINICAL* **2018**, *20*, 1148–1156, doi:10.1016/j.nicl.2018.10.002.
260. Welch, M.G.; Firestein, M.R.; Austin, J.; Hane, A.A.; Stark, R.I.; Hofer, M.A.; Garland, M.; Glickstein, S.B.; Brunelli, S.A.; Ludwig, R.J.; et al. Family Nurture Intervention in the Neonatal Intensive Care Unit Improves Social-Relatedness, Attention, and Neurodevelopment of Preterm

Infants at 18 Months in a Randomized Controlled Trial. *JOURNAL OF CHILD PSYCHOLOGY AND PSYCHIATRY* **2015**, *56*, 1202–1211, doi:10.1111/jcpp.12405.

261. Wheelock, M.D.; Lean, R.E.; Bora, S.; Melzer, T.R.; Eggebrecht, A.T.; Smyser, C.D.; Woodward, L.J. Functional Connectivity Network Disruption Underlies Domain-Specific Impairments in Attention for Children Born Very Preterm. *Cerebral Cortex* **2021**, *31*, 1383–1394, doi:10.1093/cercor/bhaa303.
262. Wheelock, M.D.; Austin, N.C.; Bora, S.; Eggebrecht, A.T.; Melzer, T.R.; Woodward, L.J.; Smyser, C.D. Altered Functional Network Connectivity Relates to Motor Development in Children Born Very Preterm. *NEUROIMAGE* **2018**, *183*, 574–583, doi:10.1016/j.neuroimage.2018.08.051.
263. Wilson-Ching, M.; Molloy, C.S.; Anderson, V.A.; Burnett, A.; Roberts, G.; Cheong, J.L.Y.; Doyle, L.W.; Anderson, P.J. Attention Difficulties in a Contemporary Geographic Cohort of Adolescents Born Extremely Preterm/Extremely Low Birth Weight. *J Int Neuropsychol Soc* **2013**, *19*, 1097–1108, doi:10.1017/S1355617713001057.
264. Wong, T.; Taylor, H.G.; Klein, N.; Espy, K.A.; Anselmo, M.G.; Minich, N.; Hack, M. Kindergarten Classroom Functioning of Extremely Preterm/Extremely Low Birth Weight Children. *EARLY HUMAN DEVELOPMENT* **2014**, *90*, 907–914, doi:10.1016/j.earlhumdev.2014.09.011.
265. Woodward, L.J.; Clark, C.A.C.; Pritchard, V.E.; Anderson, P.J.; Inder, T.E. Neonatal White Matter Abnormalities Predict Global Executive Function Impairment in Children Born Very Preterm. *DEVELOPMENTAL NEUROPSYCHOLOGY* **2011**, *36*, 22–41, doi:10.1080/87565641.2011.540530.
266. Yoneda, N.; Yoneda, S.; Tsuda, S.; Ito, M.; Shiozaki, A.; Niimi, H.; Yoshida, T.; Nakashima, A.; Saito, S. Pre-Eclampsia Complicated With Maternal Renal Dysfunction Is Associated With Poor Neurological Development at 3 Years Old in Children Born Before 34 Weeks of Gestation. *FRONTIERS IN PEDIATRICS* **2021**, *9*, doi:10.3389/fped.2021.624323.
267. You, J.; Shamsi, B.H.; Hao, M.; Cao, C.-H.; Yang, W.-Y. A Study on the Neurodevelopment Outcomes of Late Preterm Infants. *BMC NEUROLOGY* **2019**, *19*, doi:10.1186/s12883-019-1336-0.
268. Yu, Y.; Wang, T.; Liang, J.; Yang, C.; Wang, H.; Zhao, X.; Zhang, J.; Liu, W. Behavioural Problems Amongst Pre-School Children in Chongqing, China: Current Situation and Influencing Factors. *RISK MANAGEMENT AND HEALTHCARE POLICY* **2020**, *13*, 1149–1160, doi:10.2147/RMHP.S263155.
269. Zhang, L.; Chen, L.; Hong, X.; Zheng, D.; Ying, H.; Hong, L. Complete Rupture of the Pregnant Uterus: A 12-Year Retrospective Study. *INTERNATIONAL JOURNAL OF GYNECOLOGY & OBSTETRICS* **2024**, *167*, 389–394, doi:10.1002/ijgo.15576.
270. Zivan, M.; Morag, I.; Yarmolovsky, J.; Geva, R. Hyper-Reactivity to Salience Limits Social Interaction Among Infants Born Pre-Term and Infant Siblings of Children With ASD. *Front Psychiatry* **2021**, *12*, 646838, doi:10.3389/fpsyt.2021.646838.
271. Zuccarini, M.; Sansavini, A.; Iverson, J.M.; Savini, S.; Guarini, A.; Alessandroni, R.; Faldella, G.; Aureli, T. Object Engagement and Manipulation in Extremely Preterm and Full Term Infants at 6 Months of Age. *RESEARCH IN DEVELOPMENTAL DISABILITIES* **2016**, *55*, 173–184, doi:10.1016/j.ridd.2016.04.001.
